# Small-Vessel Vasculopathy Due to Aberrant Autophagy in LAMP-2 Deficiency

**DOI:** 10.1038/s41598-018-21602-8

**Published:** 2018-02-20

**Authors:** Huan T. Nguyen, Satoru Noguchi, Kazuma Sugie, Yoshiyuki Matsuo, Chuyen T. H. Nguyen, Hitoshi Koito, Ichiro Shiojima, Ichizo Nishino, Hiroyasu Tsukaguchi

**Affiliations:** 1 Second Department of Internal Medicine, Kansai Medical University, Hirakata, Osaka, Japan; 2 National Institute of Neuroscience, National Center of Neurology and Psychiatry (NCNP), Kodaira, Tokyo, Japan; 3 Department of Neurology, Nara Medical University School of Medicine, Kashihara, Nara, Japan; 4 Department of Human Stress Response Science, Institute of Biomedical Science, Kansai Medical University, Hirakata, Osaka, Japan; 5 Department of Dermatology, Kansai Medical University, Hirakata, Osaka, Japan; 6 Department of Cardiology, Otokoyama Hospital, Yawata, Kyoto, Japan

## Abstract

Lysosomal associated membrane protein 2 (*LAMP2*) is physiologically implicated in autophagy. A genetic *LAMP2* defect causes Danon disease, which consists of two major phenotypes of myopathy and cardiomyopathy. In addition, arteriopathy may manifest on rare occasions but the pathological basis remains unknown. We encountered two Danon families that developed small-vessel vasculopathy in the coronary or cerebral arteries. To investigate the underlying mechanisms, we characterized the biological features of LAMP-2–deficient mice and cultured cells. LAMP-2–deficient mice at 9–24 months of age showed medial thickening with luminal stenosis due to proliferation of vascular smooth muscle cells (VSMC) in muscular arteries. Ultrastructural analysis of VSMC revealed various autophagic vacuoles scattered throughout the cytoplasm, suggesting impaired autophagy of long-lived metabolites and degraded organelles (*i*.*e*., mitochondria). The VSMC in *Lamp2* null mice expressed more vimentin but less α-smooth muscle actin (α-SMA), indicating a switch from contractile to synthetic phenotype. Silencing of *LAMP2* in cultured human brain VSMC showed the same phenotypic transition with mitochondrial fragmentation, enhanced mitochondrial respiration, and overproduction of reactive oxygen species (ROS). These findings indicate that LAMP-2 deficiency leads to arterial medial hypertrophy with the phenotypic conversion of VSMC, resulting from age-dependent accumulation of cellular waste generated by aberrant autophagy.

## Introduction

Removal of metabolic wastes and damaged organelles such as mitochondria inside the cell is a critical process to maintain cellular homeostasis and respond to environmental stress. Macroautophagy, hereafter referred to as autophagy, is a well-studied degradation pathway that sequesters cytoplasmic cargos into double-membrane compartments (autophagosomes) and then directs them towards lysosomes^1^. Defective autophagy thus results in the accumulation of worn-out, toxic materials in tissues, thereby contributing to many human cardiovascular diseases^2,3^. LAMP-2 protein is a key molecule in the final process that mediates the fusion between autophagosomes and lysosomes. A genetic defect in *LAMP2* causes Danon disease (MIM #300257), an X-linked disorder characterized by two cardinal phenotypes of cardiomyopathy and myopathy due to excessive accumulation of autophagic vacuoles (AV) in cardiomyocytes and skeletal myofibers^4,5^. The resulting cardiac failure is a major cause of death^6,7^. Ischemic vascular disorders may occur in Danon disease on rare occasions, exemplified by cardioembolic stroke^8^ and subclinical coronary artery stenosis^9,10^. However, little clinical information is available to understand how LAMP-2 deficiency affects small arteries.

Recent studies showed that the lysosome/autophagic pathway is essential for the maintenance of vascular architecture and function^11^. Nevertheless, the mechanisms by which defective autophagy and/or storage of undegraded substrates leads to vasculopathy remain unclear. In Fabry disease, a common X-linked lysosomal storage disorder, stroke tends to occur at a significantly younger age and even higher frequency than in age-matched controls^12,13^. Pathological studies in Fabry patients showed that VSMC proliferation is induced in cerebral arteries^14^. An autophagy-related 7 knockout mouse *Atg7*^−/−^, where defective autophagy is induced conditionally only in VSMC, showed an increase in medial thickening of the aorta due to VSMC hypertrophy^15^. However, the mechanisms by which these disorders including LAMP-2 deficiency affect vascular architecture through changes in VSMC are poorly understood.

The present study aimed to clarify the clinical and biological features of vasculopathy under LAMP-2 deficiency. We first present the case of a female carrier who is heterozygous for the truncating *LAMP2* mutation and had young-onset stroke due to diffuse narrowing of the cerebral arteries without atherosclerosis. In another Danon family reported elsewhere^4,16,17^, we also re-examined the pathology of small arteries from a heterozygous mother and her hemizygous son with an exon-6 skipping mutation. The review of autopsied tissues focusing on the blood vessels revealed medial thickening of the coronary, renal, and cerebral arteries.

To further investigate the molecular basis for vasculopathy, we analyzed the pathology of the muscular arteries (cerebral, femoral, and coronary arteries) from LAMP-2–deficient mice, which recapitulate the human Danon disease. Our findings demonstrated that the VSMC are primarily responsible for medial hypertrophy, because they accumulated cytoplasmic AV and converted the phenotype from normal contractile to pathogenic, synthetic characteristics. By silencing *LAMP2* in cultured human brain VSMC *in vitro*, we found that these cells changed the phenotype towards a more proliferative state that had biological features similar to those in the vascular wall of LAMP-2–deficient mice *in vivo*. The cultured cells expressed an increase in ROS generation, mitochondrial fragmentation and enhanced mitochondrial respiration. The findings indicated that autophagy impairment and oxidative stress under LAMP-2 deficiency may accelerate the phenotypic switch of cells as an adaptive response, and this may play a critical role in the pathogenesis of medial hypertrophy.

## Results

### Small-vessel vasculopathy in patients under LAMP-2 deficiency

**Figure 1:**
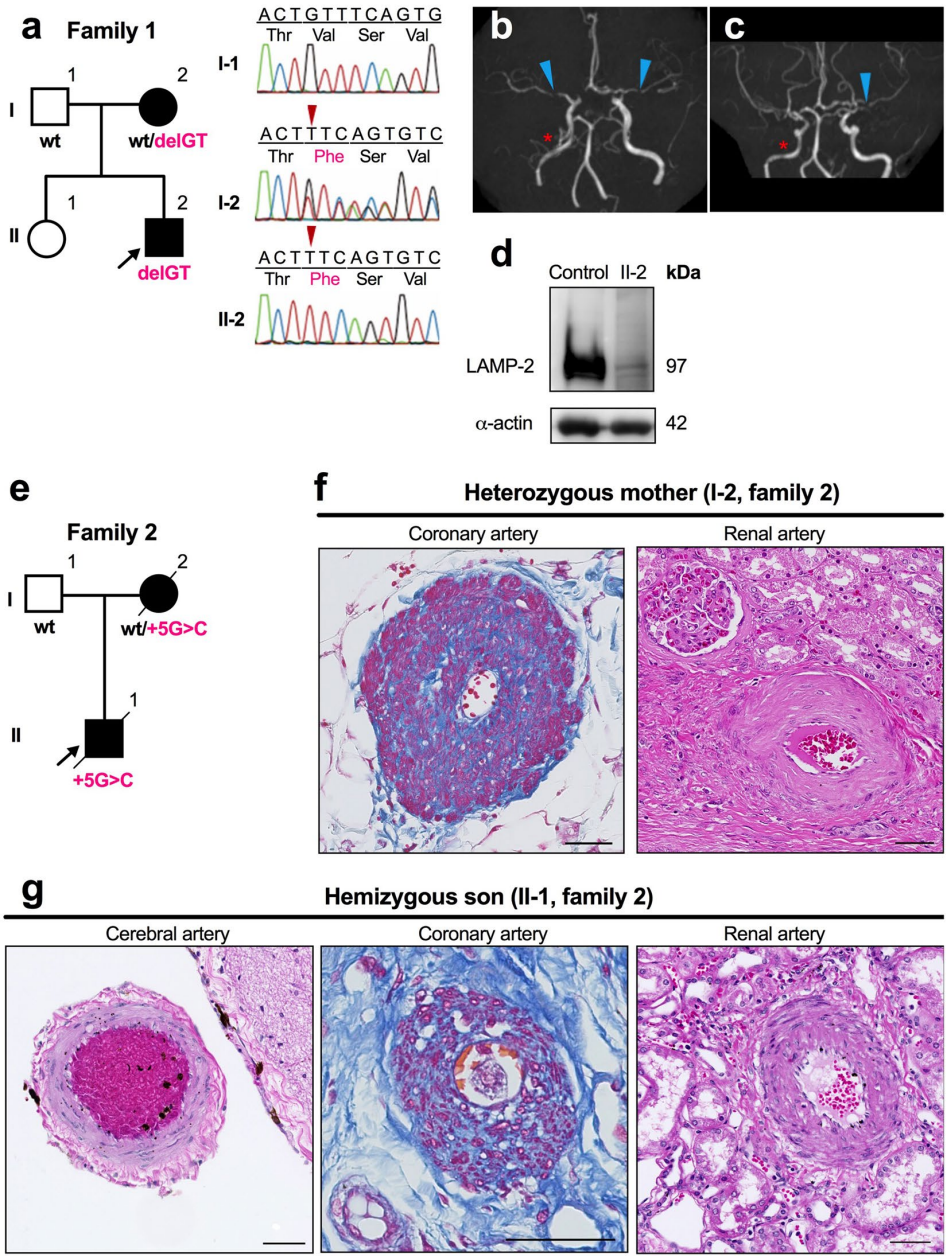
Clinical features and genetics of LAMP-2–deficient patients. (**a**) Pedigree and *LAMP-2* mutations in the first family. The affected son (II-2) was hemizygous for c.1009_1010 delGT: p.Val337Phe fs*12 (delGT), while the mother (I-2) was heterozygous. *Arrowheads* in the chromatograms indicate the position of the deletion. wt, wild-type. (**b**,**c**) Brain MRA images in the affected mother at the age of 47 years (B, axial view; C, coronal view) are shown. She exhibited a bilateral diffuse narrowing of the middle cerebral artery *(arrowheads)*. There was also mild, irregular stenosis in the proximal portion of the right internal carotid artery (*asterisks*). (**d**) Western blot analysis of skeletal muscle in the affected son (II-2). LAMP-2 is abundantly expressed in control muscle, whereas only trace levels were found in the patient’s muscle. (**e**) Pedigree of the second family. A splice-donor site mutation (c.864 + 5 G >C, in intron 6) causes an in-frame exon-6 skipping (+5 G > C); genotypes are shown below the symbols. Light microscopy showed thickened media in muscular arteries of both the mother (**f**) and her son (**g**). Brain autopsy was not performed in the mother. Azan staining was used for the coronary arteries, whereas H&E staining was used for the renal and cerebral arteries. Scale bars: 50 µm.

In the first family, a 50-year-old female (I-2) (Fig. 1a) developed ischemic stroke with transient left hemiparesis at the age of 47, which was successfully treated by by-pass surgery. Brain magnetic resonance angiography (MRA) showed a bilateral, diffuse narrowing of the cerebral arteries as well as some irregular stenosis of right internal carotid artery (Fig. 1b,c). She did not have any clinical risk factors for atherosclerosis, including hypertension, smoking, or dyslipidemia, nor was there any obvious stenosis in the aorta or its branches (Supplementary Fig. S4). Cardiac imaging studies revealed hypertrophic cardiomyopathy (HCM) but no intracardiac thrombus (Supplementary Fig. S5a). Her 16-year-old son (II-2) sought medical attention for muscle weakness, liver dysfunction, and mild intellectual disability. Muscle biopsy demonstrated numerous AV among the myofibrils (Supplementary Fig. S6). He had HCM similar to that in his mother, but he lacked any stenosis of the cerebral arteries (Supplementary Fig. S1b). These clinical features raised the possibility of Danon disease. Sequence analysis of the *LAMP2* gene (NM_013995.2) disclosed that the mother is heterozygous for a novel frameshift mutation (c.1009_1010 delGT), while her affected son is hemizygous (Fig. 1a). This mutation is predicted to delete the C-terminal transmembrane spanning domain of the LAMP-2 protein encoded by the last coding exon 9, which serves as the lysosomal targeting signal. Western analysis with a skeletal muscle biopsy specimen from the affected son revealed that the truncated LAMP-2 protein was barely expressed (Fig. 1d). These molecular studies confirmed the diagnosis of Danon disease in this family.

In the second Danon family (Fig. 1e), the proband boy had a hemizygous exon-6-skipping mutation in *LAMP2* (Nishino I, *et al*., patient 3)^4^, while the mother was heterozygous for that variant. He manifested dilated cardiomyopathy at the age of 22 and he died from cardiac failure at the age of 31. Besides cardiomyopathy, his mother suffered from liver disease and died of heart failure at the age of 52. Re-examination of the autopsied tissues for pathology showed thickened media in their muscular arteries (Fig. 1f,g).

### Histological analysis of arterial vasculopathy in LAMP-2–deficient mice

**Figure 2:**
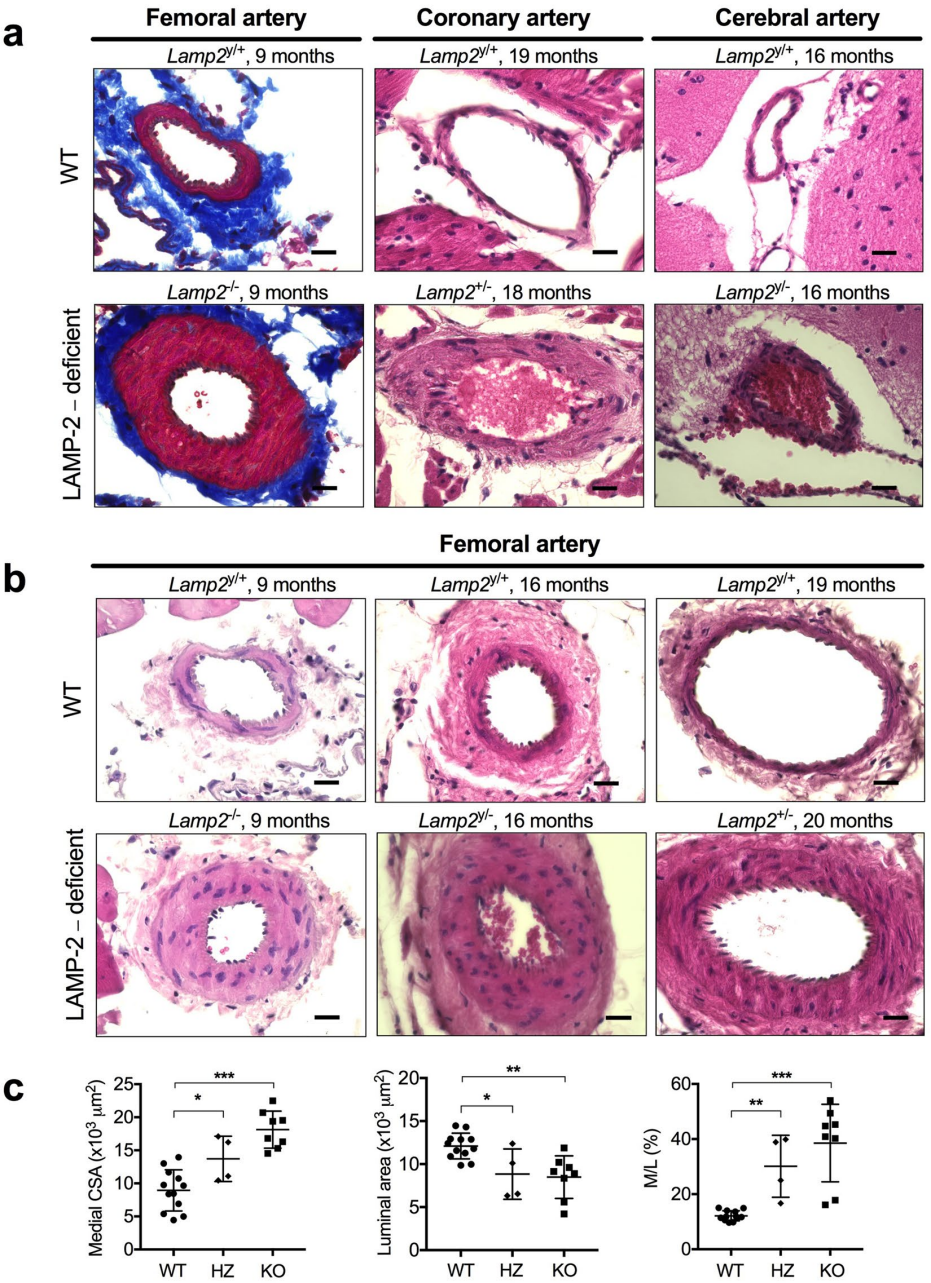
Histopathology of muscular arteries in LAMP-2–deficient mice. (**a**) Light microscopy showed a thickened media in the femoral, coronary, and cerebral arteries of LAMP-2–deficient mice, but not in age-matched wild-type mice. The femoral arteries were stained with Masson’s trichrome; the coronary and cerebral arteries were stained with H&E. Scale bars: 20 µm. (**b**) Medial thickening with a narrowed lumen is seen in *Lamp2* null mice (female homozygosity *Lamp2*^−/−^, and male hemizygosity *Lamp2*^y/−^ as well as female heterozygosity *Lamp2*^+/−^), but not in age-matched wild-type mice (*Lamp2*^y/+^). H&E staining. Scale bars: 20 µm. (**c**) Morphometric assessment of femoral arteries showed a significant increase in medial CSA, decrease in luminal area, and increase in the media thickness-to-lumen diameter ratio (M/L) in *Lamp2* KO mice. *P < 0.05, **P < 0.01, and ***P < 0.001, compared with *Lamp2* WT mice. A two-tailed *one-way ANOVA* was used followed by *Tukey’s* post-hoc test. *n* = 12, 4, and 8 for the WT, HZ, and KO groups, respectively. HZ, heterozygous; KO, knockout; WT, wild-type.

The observations above suggest that defective autophagy due to LAMP-2 deficiency may be related to the pathogenesis of narrowed arteries in two families. To further address this issue, we studied the pathology and molecular features of muscular arteries in LAMP-2–deficient mice^5^. We chose mice with an age ranging from 9 to 24 months (*n* = 12) so that we could evaluate the possible effects of aging. We examined the pathology of three types of muscular arteries: femoral, coronary, and cerebral arteries. Light microscopy revealed that the vascular walls were thickened in LAMP-2–deficient mice, either in the null state (*Lamp2*^−/−^ or *Lamp2*^y/−^) or in heterozygosity (*Lamp2*^+/−^), but not in wild-type mice (*Lamp2*^y/+^ or *Lamp2*^+/+^). Masson trichrome staining of femoral arteries revealed the presence of arterial medial hypertrophy, primarily arising from VSMC proliferation, and there were no apparent changes in endothelial cells (Fig. 2a,b).

Morphometric analysis of the femoral arteries demonstrated that the medial cross-sectional area (CSA) was significantly greater in *Lamp2* knockout mice than in wild-type mice (18.1 ± 2.8 × 10^3^ µm^2^ vs. 8.9 ± 3.1 × 10^3^ µm^2^ in wild-type, *P* < 0.001, *n* = 8 and *n* = 12 for the knockout and wild-type groups, respectively). The luminal area of the femoral arteries of *Lamp2* knockout mice was significantly smaller than that of wild-type mice (8.4 ± 2.4 × 10^3^ µm^2^ vs 12.1 ± 1.5 × 10^3^ µm^2^ in wild-type, *P* < 0.01, *n* = 8 and *n* = 12 for the knockout and wild-type groups, respectively). The tendency for hypertrophic alteration was also suggested by the age-matched heterozygous *Lamp2* mice (Fig. 2c and Supplementary Table S3). The data indicate that LAMP-2 deficiency leads to hypertrophic remodeling with medial thickening, thereby resulting in small-vessel disease^18^.

### Impaired autophagic flux in LAMP-2–deficient VSMC

**Figure 3:**
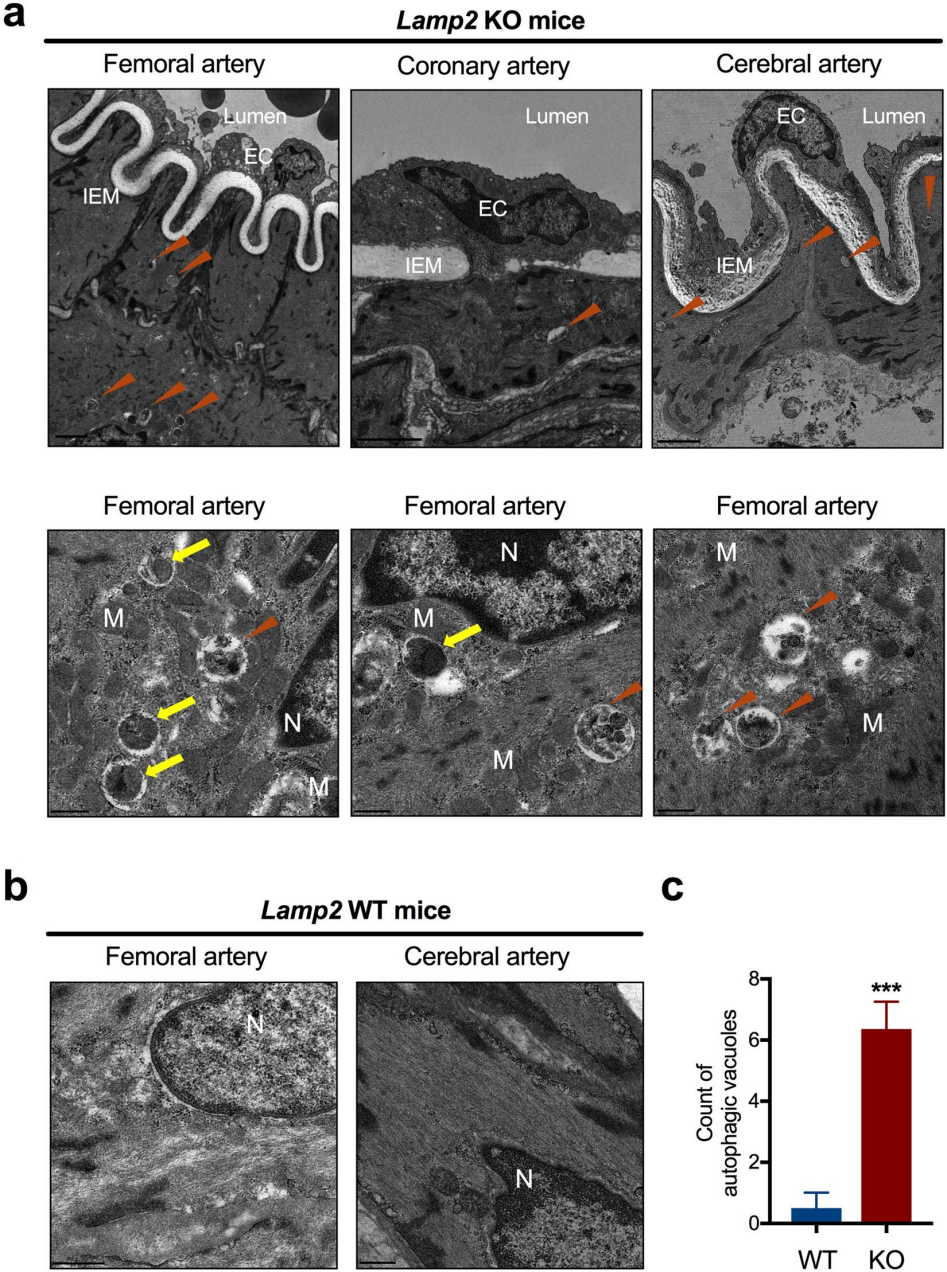
Ultrastructure of AV in the media of muscular arteries in *Lamp2* KO mice. (**a**) *(Upper panels*) Low-magnification electron microscopy of the intimal and medial border. Vacuoles were accumulated in the cytoplasm of VSMC in femoral, coronary, and cerebral arteries *(arrowheads)*. Scale bars: 2 µm. *(Lower panels)* Higher magnification for vacuolar structures in the media of femoral arteries. VSMC contained AV in the cytoplasm *(arrowheads)* with various contents, some of which were autophagosomes containing morphologically abnormal mitochondria *(arrows*). Scale bars: 0.5 µm. (**b**) Cytoplasm of VSMC from *Lamp2* WT mice. Scale bars: 0.5 µm. (**c**) Quantitative data were obtained from 10 distinctive 4.25-µm square sections, randomly chosen from the cytoplasm of VSMC. The number of AV in VSMC comprising the media of muscular arteries was significantly greater in *Lamp2* KO mice than in *Lamp2* WT mice. ***P < 0.001. A two-tailed *Student’s t*-test was used. EC, endothelial cells; IEM, internal elastic membrane; KO, knockout; M, mitochondria; N, nucleus; WT, wild-type.

**Figure 4:**
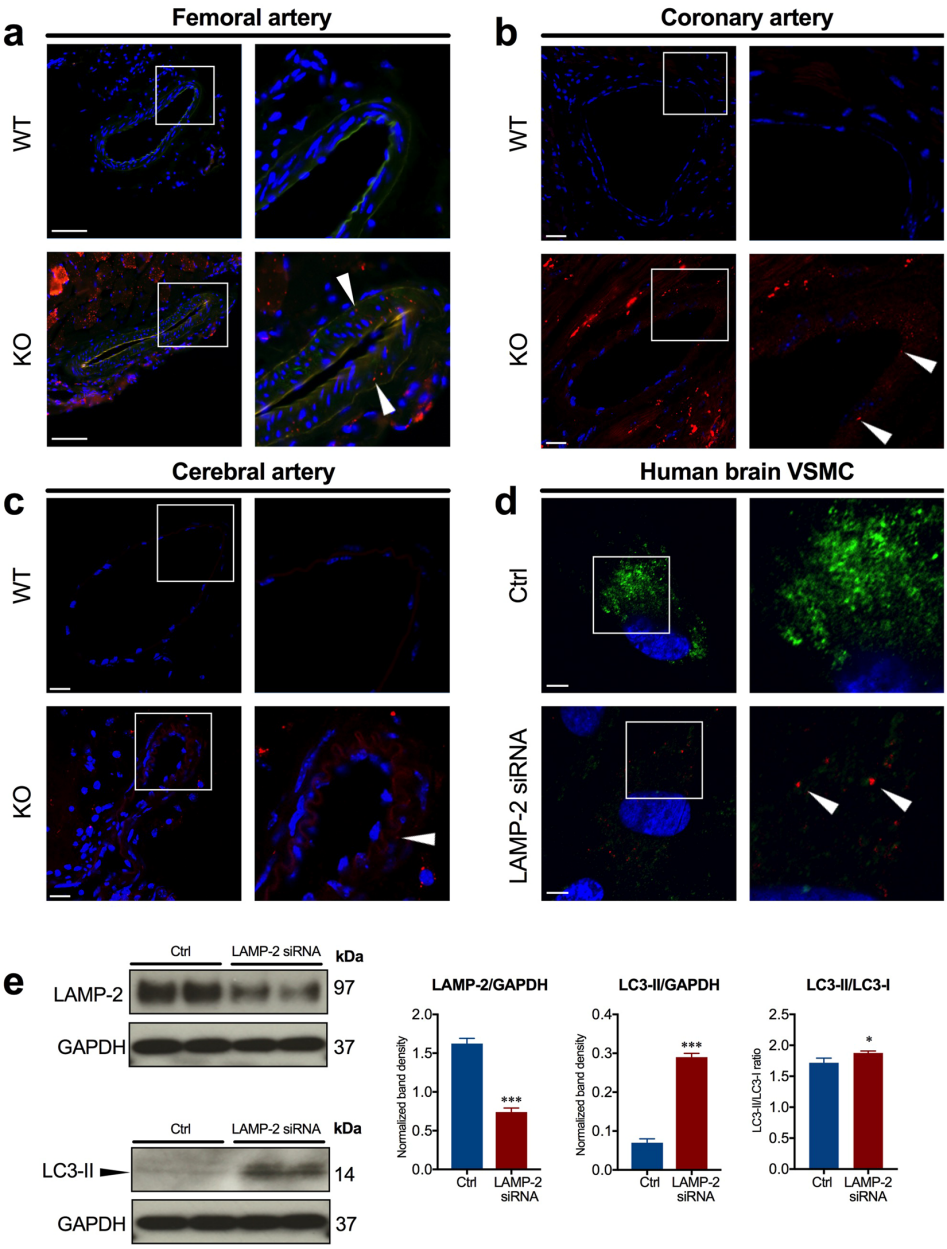
Accumulation of autophagosomes in the media of muscular arteries in *Lamp2* KO mice and cytoplasm of cultured human brain VSMC in LAMP-2 deficiency. Appearance of autophagosomes visualized by LC3 *(red) (arrowheads)* in the media of the femoral (**a**), coronary (**b**), and cerebral (**c**) arteries in *Lamp2* KO mice. Nuclei are counterstained by DAPI *(blue)*. Staining of α-SMA showed the localization of VSMC in the media of femoral arteries *(green* in a*)* (*n* = 4 per group). Scale bars: 100 µm. (**d**) LC3 staining in cultured human brain VSMC of control or LAMP-2 siRNA-transfected cells showed accumulation of autophagosomes *(red) (arrowheads)* in the cytoplasm of cells under LAMP-2 deficiency. Successful silencing of LAMP-2 RNA via siRNA treatment was proven by the attenuation of cytoplasmic LAMP-2 signals *(green)*. Nuclei are counterstained by DAPI *(blue)*. Scale bars: 5 µm. (**e**) Western blot analysis of LAMP-2 and LC3 in cultured human brain VSMC. LAMP-2 siRNA-treated vs control groups are compared. Quantified data from 3 independent experiments are shown. ***P < 0.001. A two-tailed *Student’s t*-test was used. Ctrl, control; KO, knockout; WT, wild-type.

To further understand the molecular mechanisms of medial thickening, we examined the ultrastructure of medial VSMC in muscular arteries of *Lamp2* knockout mice. Electron microscopy with VSMC in the femoral, coronary, and cerebral arteries showed accumulation of AV with polymorphous contents such as fragmented cellular organelles and/or granular materials. Some vacuoles in VSMC contained undigested mitochondrial fragments, suggesting aberrant mitophagy. Notably, the vacuoles were absent from vascular endothelial cells (Fig. 3). Double immunostaining with the autophagosome marker, microtubule-associated protein 1 light chain 3 (LC3), and α-SMA showed an increase in LC3 puncta in the medial VSMC of muscular arteries in *Lamp2* knockout mice, compared with those of the wild-type (Fig. 4a–c). Treatment of human brain VSMC with LAMP-2 siRNA increased the number of LC3-positive puncta in cytoplasm and the LC3 protein abundancy in Western analysis (Fig. 4d,e). These results suggest that cytoplasmic vacuoles structurally and functionally appear to be autophagosomes.

**Figure 5:**
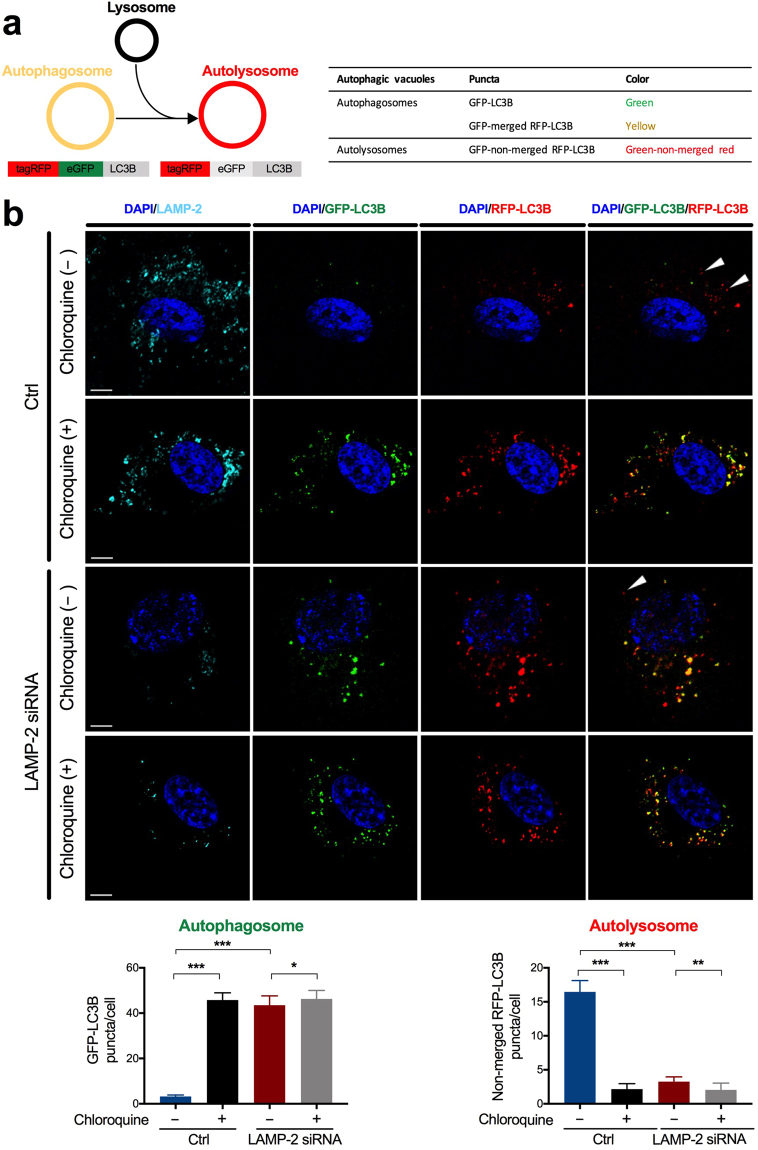
Impaired autophagic flux in LAMP-2–deficient human brain VSMC. (**a**) Fluorescent visualization of autophagosomes *(GFP-positive puncta*, *green or yellow)* and autolysosomes (*GFP-non-merged RFP-positive puncta*, *green-non-merged red)* by the use of the autophagy tandem sensor RFP-GFP-LC3B. (**b**) Compared with control *(top panel)*, chloroquine alone *(second panel)*, LAMP-2 siRNA alone *(third panel)*, and double treatment *(bottom)* showed an increase in autophagosomes with an inverse reduction in autolysosomes *(arrowheads)*. *P < 0.05, **P < 0.01, and ***P < 0.001. The nuclei and LAMP-2 protein were stained by DAPI *(blue)* and LAMP-2 *(turquoise)*, respectively. Scale bars: 5 µm. A two-tailed *one-way ANOVA* was used followed by *Tukey’s* post-hoc test for all panels.

To monitor autophagic flux in VSMC, we employed the technique of tandem sensor Red Fluorescent Protein-Green Fluorescent Protein (RFP-GFP)-LC3B under chloroquine administration or siRNA suppression. In this assay, autophagosomes are visualized as green or yellow (GFP-positive puncta), whereas autolysosomes are labeled as green-non-merged red (GFP-non-merged RFP-positive puncta) (Fig. 5a). Chloroquine treatment of control VSMC resulted in a significant increment in the accumulation of autophagosomes, suggesting the existence of endogenous, basal autophagic flux^19,20^ in VSMC (number of GFP-LC3B puncta per cell: 45.8 ± 3.2 in chloroquine-treated vs. 3.2 ± 0.7 in untreated control, *P* < 0.001). When transfected with LAMP-2 siRNA, cells yielded a greater number of autophagosomes (number of GFP-LC3B puncta per cell: 43.5 ± 4.1 in LAMP-2 siRNA-treated vs. 3.2 ± 0.7 in scramble siRNA-treated control, *P* < 0.001). Conversely, the number of autolysosomes was significantly reduced under LAMP-2 deficiency (number of GFP-non-merged RFP-LC3B puncta per cell: 3.3 ± 0.7 in LAMP-2 siRNA-treated vs. 16.5 ± 1.7 in scramble siRNA-treated control, *P* < 0.001) (Fig. 5b). The fewer autolysosomes support our notion that LAMP-2 deficiency blocks the autophagosome-lysosome fusion step, but does not stimulate the formation of autophagosomes. Taken together, our observations indicate that autophagy flux is constitutively active in VSMC to some extent under normal conditions, but is more markedly impaired by blockage at the fusion step under a LAMP-2–deficient state.

### Phenotypic conversion of VSMC in LAMP-2 deficiency

**Figure 6:**
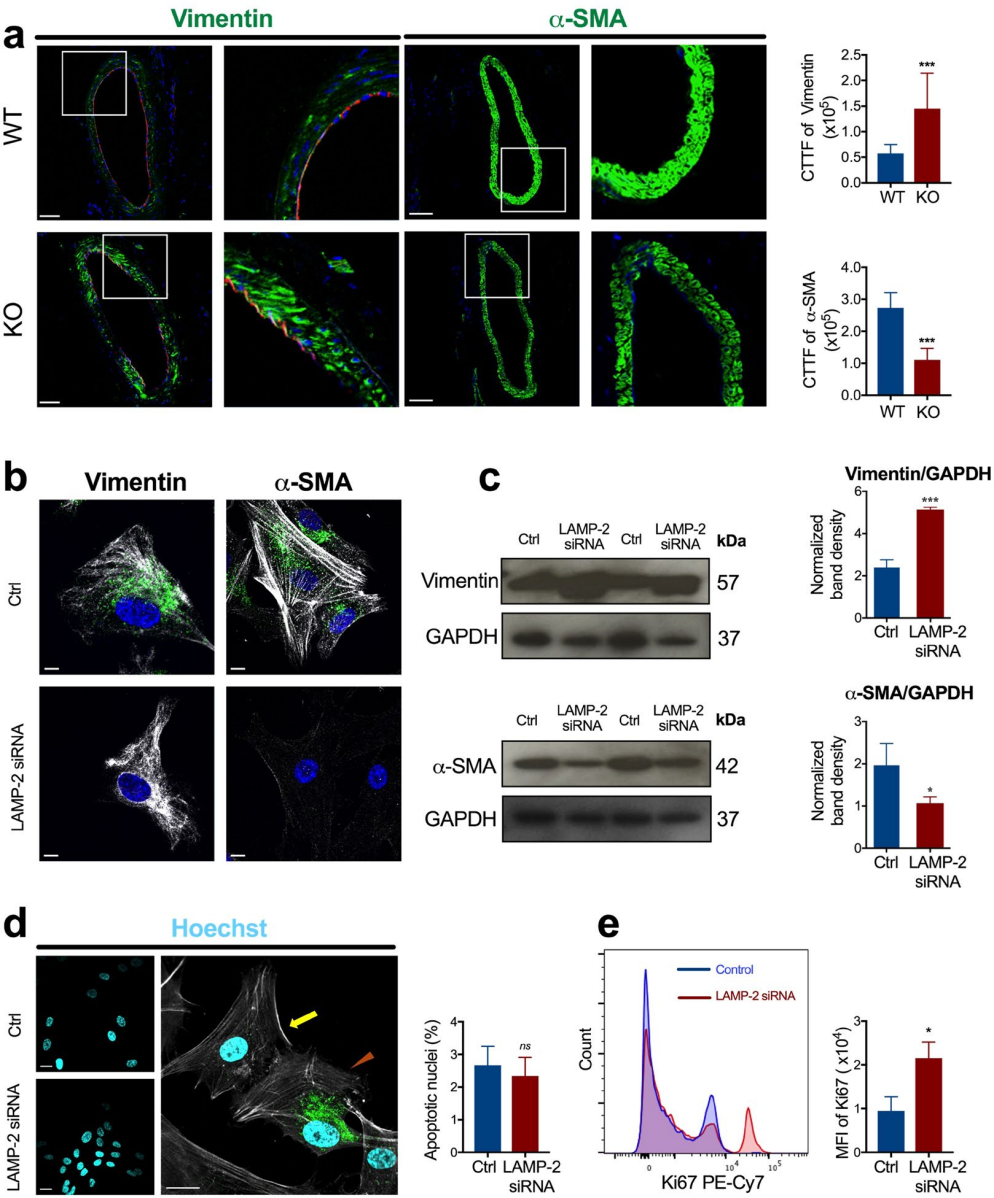
Morphologic and functional characteristics of VSMC under LAMP-2 deficiency. (**a**) Immunostaining of VSMC in the femoral arteries of *Lamp2* KO and WT mice. In *Lamp2* KO mice, the synthetic marker vimentin was increased (*green*), whereas the contractile marker α-SMA was conversely reduced *(green)*. Endothelial cells were stained by CD31 *(red)*, and nuclei were visualized by DAPI *(blue)*. Scale bars: 50 µm. The graphs show quantitation of the vascular markers from the immunostaining. The media of *Lamp2* KO mice expressed more vimentin but less α-SMA than the media of WT mice. ***P < 0.001, compared with *Lamp2* WT mice (*n* = 4 per group). (**b**) Phenotypic switch of LAMP-2–deficient human brain VSMC. The LAMP-2 mRNA suppression led to increased vimentin expression *(white)* with an inverse reduction in α-SMA *(white)*, compared with control cells. The nuclei and cytoplasmic LAMP-2 protein were stained by DAPI *(blue)* and LAMP-2 *(green)*, respectively. Scale bars: 10 µm. (**c**) Western blot analysis of vimentin and α-SMA protein in LAMP-2 siRNA-treated human brain VSMC vs control cells. The graphs show quantified data from the Western blots. *P < 0.05, ***P < 0.001, compared with control cells. (**d**) Evaluation of apoptosis *(left upper and lower panels)*. Nuclei of LAMP-2 siRNA treated and control cells were stained with Hoechst 33342 *(turquoise)*. Both subgroups showed a normal nuclear appearance *(right panel)*. The actin filaments and LAMP-2 protein were stained by Phalloidin *(white)* and LAMP-2 *(green)*, respectively. The morphological features of nuclei appeared normal in both siRNA-treated *(arrow)* and control cells *(arrowhead)*. Scale bars: 20 µm. There was no difference in the frequency of apoptotic cells between the two groups. *ns*, not significant. (**e**) Proliferation activity was assessed by detection of Ki67-positive cells using flow cytometery. Histograms show an increased Ki67-positive population in LAMP-2 siRNA-treated human brain VSMC, compared with control cells. The bar graph data were obtained from 3 independent experiments. **P < 0.01. A two-tailed *Student’s t*-test was used for all panels. Ctrl, control; CTTF, corrected total tissue fluorescence; KO, knockout; MFI, mean fluorescence intensity; WT, wild-type.

To further investigate the biological characteristics of VSMC, we examined their morphological and functional aspects in LAMP-2–deficient mice and cells. VSMC exhibit a normal contractile phenotype at steady state but converts into a synthetic (proliferative) phenotype under various stress conditions. In *Lamp2* knockout mice, immunostaining of medial VSMC from the femoral arteries showed a marked increase in the synthetic marker vimentin with an inverse reduction of the contractile marker α-SMA (Fig. 6a). LAMP-2 mRNA suppression in cultured human brain VSMC led to similar phenotypic changes (Fig. 6b,c). The data indicate that a switch from contractile to synthetic phenotype occurs in both muscular arteries as well as cultured human brain VSMC under a LAMP-2–deficient state.

### Apoptosis and proliferation of VSMC in LAMP-2 deficiency

To further evaluate the cellular effects of LAMP-2 deficiency in VSMC, we next examined apoptosis and proliferation activity in LAMP-2 siRNA-treated human brain VSMC using Hoechst 33342 and Ki67 markers, respectively. We found no significant difference in the number of apoptotic cells between LAMP-2–deficient and control cells (% of apoptotic cells: 2.3 ± 0.3 vs. 2.7 ± 0.3 in control) (Fig. 6d). FACS analysis revealed the suppression of LAMP-2 conversely leads to an increment in proliferative cells, which were labeled by positive staining with Ki-67 (mean fluorescence intensity: 2.16 ± 0.21 × 10^4^ vs. 0.95 ± 0.19 × 10^4^ in control, *P* < 0.001) (Fig. 6e). The findings with cultured cells indicate that LAMP-2 deficiency promotes proliferation without inducing any apoptosis of VSMC. This phenotypic switching of VSMC from contractile to proliferative state in LAMP-2–deficiency may account for the medial hypertrophy found in muscular arteries of humans and mice (Figs 1f,g and 2).

### Increased mitochondrial fragmentation and oxidative stress in LAMP-2–deficient VSMC

**Figure 7:**
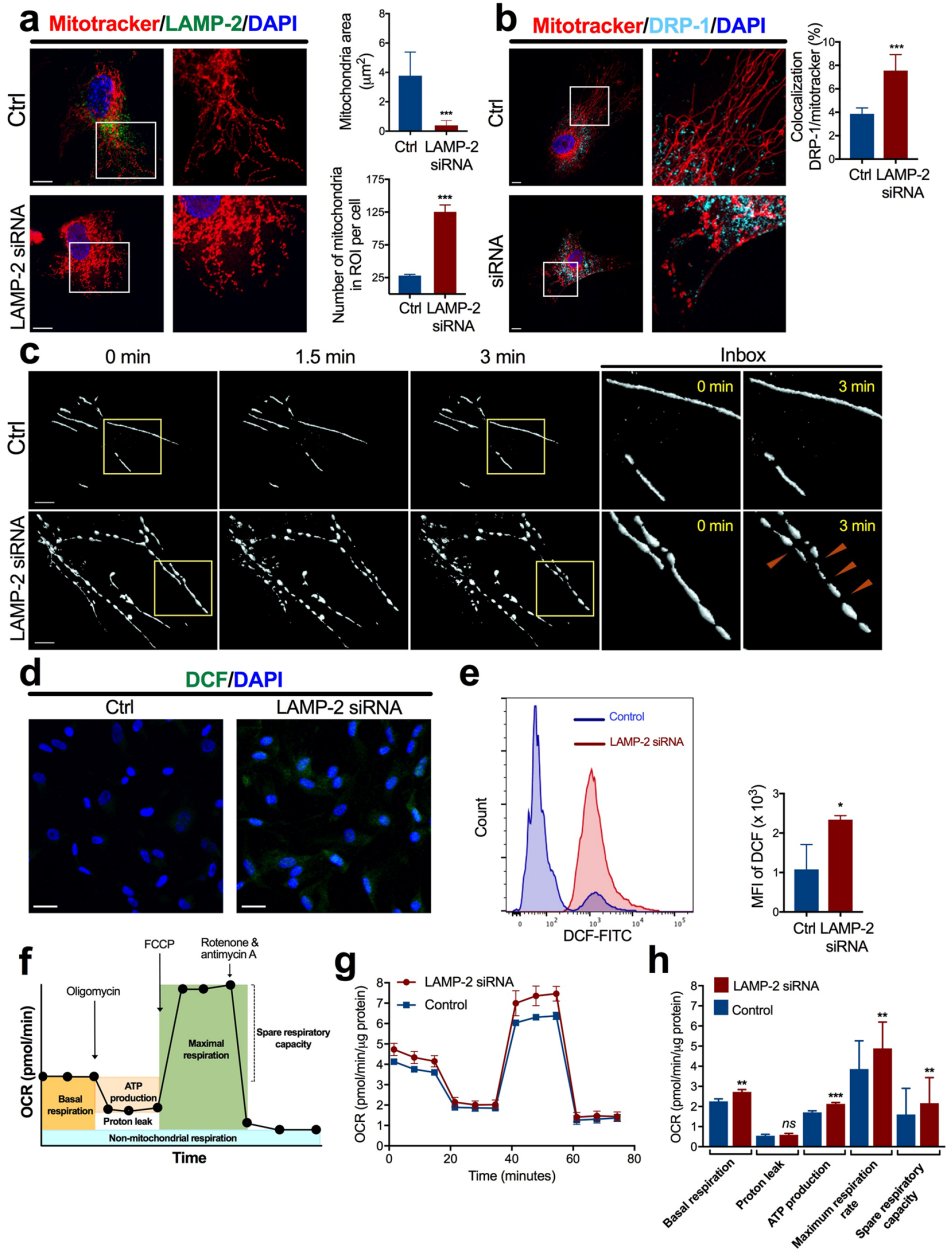
Alterations in mitochondrial dynamics and function of VSMC under LAMP-2 deficiency. (**a**) Morphology of mitochondria in live VSMC. Mitotracker staining *(red)* revealed more marked fragmentation of mitochondria in LAMP-2 siRNA human brain VSMC, compared with control cells. The nuclei and cytoplasmic LAMP-2 protein were stained by DAPI *(blue)* and LAMP-2 *(green)*, respectively. Scale bars: 10 µm. The graphs show that LAMP-2 deficiency induced significantly decreased individual mitochondrial area and significantly increased number of mitochondria in ROI per cell. ***P < 0.001, compared with control cells. (**b**) Double immunostaining of DRP-1 (*turquoise*) and Mitotracker (*red*). The nuclei were visualized by DAPI *(blue)*. The colocalization of DRP-1 protein and mitochondria is indicated by *white*. Scale bars: 10 µm. Colocalization of DRP-1 with MitoTracker is more obvious in VSMC under LAMP-2 deficiency, compared with control cells. ***P < 0.001. (**c**) 3D time-lapse reconstructed images of mitochondria in live VSMC. Time-lapse tracking during 3 minutes revealed that mitochondria in LAMP-2–deficient VSMC changed their shape to become smaller, swollen, and more spherical, thereby resulting in a fragmented pattern (*arrowheads*). In contrast, control cells maintained normal mitochondrial dynamics, with fine tubular networks. Scale bars: 5 µm. (**d**) Immunocytochemical detection of H_2_O_2_ in LAMP-2 siRNA human brain VSMC. *Green fluorescence* indicates DCF production. Nuclei were visualized by DAPI *(blue)*. Bars: 50 µm. (**e**) H_2_O_2_ content was evaluated in flow cytometry analysis by quantification of DCF. Histograms showed enhanced DCF expression in LAMP-2 siRNA-treated human brain VSMC, compared with control cells. Quantified data from 3 independent experiments are shown. *P < 0.05. (**f**) Mitochondrial respiration assay. Oligomycin, FCCP, and a mix of rotenone/antimycin A were sequentially added to measure basal respiration, ATP production, proton leak, maximal respiration, spare respiratory capacity, and non-mitochondrial respiration. (**g**) Representative line graphs of mitochondrial respiration assays are shown. (**h**) LAMP-2 siRNA human brain VSMC showed a significant increase in mitochondrial respiration. *P < 0.05, **P < 0.01, ***P < 0.001, and *ns*, not significant, compared with control cells. A two-tailed *Student’s t*-test was used for all panels. Ctrl, control; MFI, mean fluorescence intensity.

Since aberrant mitophagy was suggested by ultrastructural studies with VSMC in LAMP-2–deficient mice, we next investigated mitochondrial dynamics in cultured cells under both scramble siRNA-treated and LAMP-2 siRNA-treated conditions. We performed double immunostaining with MitoTracker and DRP-1, a GTPase necessary for initiation of mitochondrial division. The results revealed that LAMP-2 deficiency induced a remarkable mitochondrial morphologic abnormality as well as a change in subcellular distribution with the accumulation of fragmented, swollen, spherical organelles in the perinuclear region. In contrast, control cells showed fine, straight tubular networks, which were evenly spread throughout the cytoplasm. After silencing LAMP-2 expression, punctate staining of DRP-1 became more colocalized with the fragmented mitochondria (% of colocalization: 7.6 ± 0.3 vs. 3.9 ± 0.1 in control, *P* < 0.001) (Fig. 7a,b).

To further investigate mitochondrial dynamics, we performed time-lapse confocal imaging of mitochondria in live VSMC, which were labelled with MitoTracker. During 3 minutes of time-lapse tracking, the 3D reconstructed images revealed that mitochondria changed their shape to become smaller, swollen, and more spherical in LAMP-2–deficient VSMC. In control cells, the mitochondria showed fine tubular networks (Fig. 7c). Taken together, our observations indicate that LAMP-2 deficiency accelerated mitochondrial fission, thereby enhancing fragmentation.

To estimate ROS production, we measured total intracellular H_2_O_2_ using the probe H_2_DCF-DA. When LAMP-2 was silenced by siRNA, human brain VSMC showed greater H_2_O_2_ levels compared with control cells (Fig. 7d,e). The results indicate that LAMP-2 deficiency results in greater oxidative stress of VSMC due to ROS accumulation that is partly mediated by aberrant mitophagy.

### Enhanced respiration rate in LAMP-2–deficient VSMC

In view of the dysmorphic mitochondria in LAMP-2–deficient VSMC, we next evaluated the function of mitochondria. For this purpose, we measured the oxygen consumption rate (OCR) in cultured human brain VSMC. The results revealed that LAMP-2–deficient human brain VSMC showed an enhanced mitochondrial respiration, compared with that in control cells. The basal respiration, ATP production, maximal respiratory rate, and spare respiratory capacity were all significantly increased (Fig. 7f–h). The data indicate that a reduced LAMP-2 level is functionally related to greater oxygen consumption.

## Discussion

Small-vessel diseases (e.g., cerebral arteries) have a significant impact on morbidity and mortality. However, the mechanisms remain largely elusive^21^. Association of small-vessel vasculopathy with LAMP-2 deficiency has been suggested by two previous findings in the literature: medial thickening of coronary arteries was observed in individuals who had a truncating mutation in both the hemizygous (p.Tyr109ter)^9^ and heterozygous (p.Phe151 fs*32)^10^ state. In the present study, we first found that the heterozygous mother, who harbored a frameshift mutation p.Val337Phe fs*12, developed young-onset stroke due to cerebral artery stenosis (family 1). In another family (family 2) with in-frame deletion of exon 6, postmortem histological analyses further revealed medial thickening of the coronary and renal arteries in the heterozygous carrier mother and hemizygous affected son. These observations together with others suggest that small-vessel vasculopathy could develop in individuals with LAMP-2 deficiency under some circumstances.

We assume that at least three factors contribute to the pathogenesis of vasculopathy under LAMP-2 deficiency. First, in heterozygous female carriers with the *LAMP2* null allele, non-randomized inactivation of X-chromosome might have occurred so that the mesenchymal progenitor cells differentiating into vascular components have a preferentially higher content of mutant cells than other lineages. This X-chromosome inactivation usually operates at a very early embryonic stage; thus, the extent of skewed expression of the wild-type vs mutant varies widely among tissues as well as individuals^22^. In agreement with this notion, there have been reports of female careers who required heart transplantation due to severe cardiomyopathy^7,10^.

Second, aging may modify the structure and function of the vessel walls, given that both autophagy and mitochondrial functions generally decline with age^23–25^. In accordance with this notion, the affected son in family 1, who lacked functioning LAMP-2, did not show any clinical vasculopathy. The absence of vascular disease is in sharp contrast to his mother, who had prominent narrowing of the cerebral arteries. A decline in autophagy flux with age leads to the accumulation of long-lived proteins and damaged organelles (i.e., mitochondria) and further increases in oxidative stress. These interdependent processes will synergistically act on medial VSMC and exceed a certain threshold that leads to clinical pathology. A longer follow-up of Danon patients and obligate carriers is required to address the mechanisms by which aging contributes to the pathogenesis of vascular injuries.

Third, the relative expression of LAMP-1 vs LAMP-2 as well as lysosome enzyme contents (*i*.*e*., Cathepsin D, *etc*.) may differ among tissues as well as individuals^26^. Compensatory upregulation of LAMP-1 might occur in some tissues in a LAMP-2–deficient state^27,28^. Thus, tissue-specific variability in relative LAMP protein abundance may modulate the vascular phenotypes; the tissues in which alternate LAMP-1 upregulation can not compensate for the defective LAMP-2 in VMSC are more susceptible to injury leading to vasculopathy.

In the present study, our data revealed that medial thickening occurs in *Lamp2* null mice. Careful evaluation of female *Lamp2* heterozygous mice suggests that arterial stenosis may develop in the heterozygous state under some conditions. Consistent with this view, previous studies on the hearts of *Lamp2* heterozygous mice showed a marginal functional abnormality of the myocardium (reduced left ventricular ejection) with a significant increase in autophagosomes in the myocardium at 8 weeks of age, as compared with wild-type females^29^. These observations together with ours suggest that a 50% reduction in *Lamp2* gene dosage in female heterozygous *Lamp2*^+/−^ mice does not normally cause any overt abnormalities. However, tissue damage may occur with age when the *Lamp2* dosage is reduced by 50% and could become overt under some circumstances: (1) the presence of skewed X-chromosome inactivation^22^, (2) coexisting environmental risk factors and/or changes in female hormones, or (3) tissue-specific LAMP-1 vs LAMP-2 compensation^27^.

Our experimental results with mice and cultured cell revealed that LAMP-2 deficiency can lead to medial hypertrophy, mainly through primary morphological changes in VSMC. The smooth muscle cells that comprise the arterial walls usually do not show active proliferation. However, once the cellular stress exceeds a threshold, the VSMC convert to a synthetic state so that they could adapt to the environment by remodeling of vascular structures^30^. A previous study demonstrated that LAMP-1/2 double-deficient mice showed a much higher frequency of cytoplasmic AV in vascular endothelial cells^31^. In contrast, we did not found any AV in the endothelial cells of muscular arteries in *Lamp2* knockout mice, suggesting that VSMC may be more vulnerable to defective autophagy than endothelial cells. This presumably reflects an important difference in the relative abundance of LAMP-1 vs LAMP-2, and/or lysosomal enzyme contents. Furthermore, the capability of VSMC to undergo mitosis may account for the increased susceptibility to undigested toxic materials as well as oxidative injury, and they are prone to more oxidative stress when they develop a proliferative state^32^. In contrast, cardiomyocyte and skeletal myofibers, which are post-mitotic cells in a senescent state, may respond in a different way so they undergo degeneration rather than proliferation^33^ (Supplementary Fig. S9).

The role of autophagy in vascular disease is a critical matter of debate^2,34^. Our data indicate that impaired autophagy is significantly implicated in the medial thickening of muscular arteries in LAMP-2 deficiency. The relevance of autophagy in vascular disorders is also supported by previous studies showing that there is thickened aortic media along with cellular hypertrophy in VMSC-specific *Atg7* knockout mice. Further study is needed to understand how VSMC could behave differently in response to a deficiency of earlier-phase mediators (i.e., *Atg7)* vs late- phase mediators (LAMPs)^15^.

**Figure 8:**
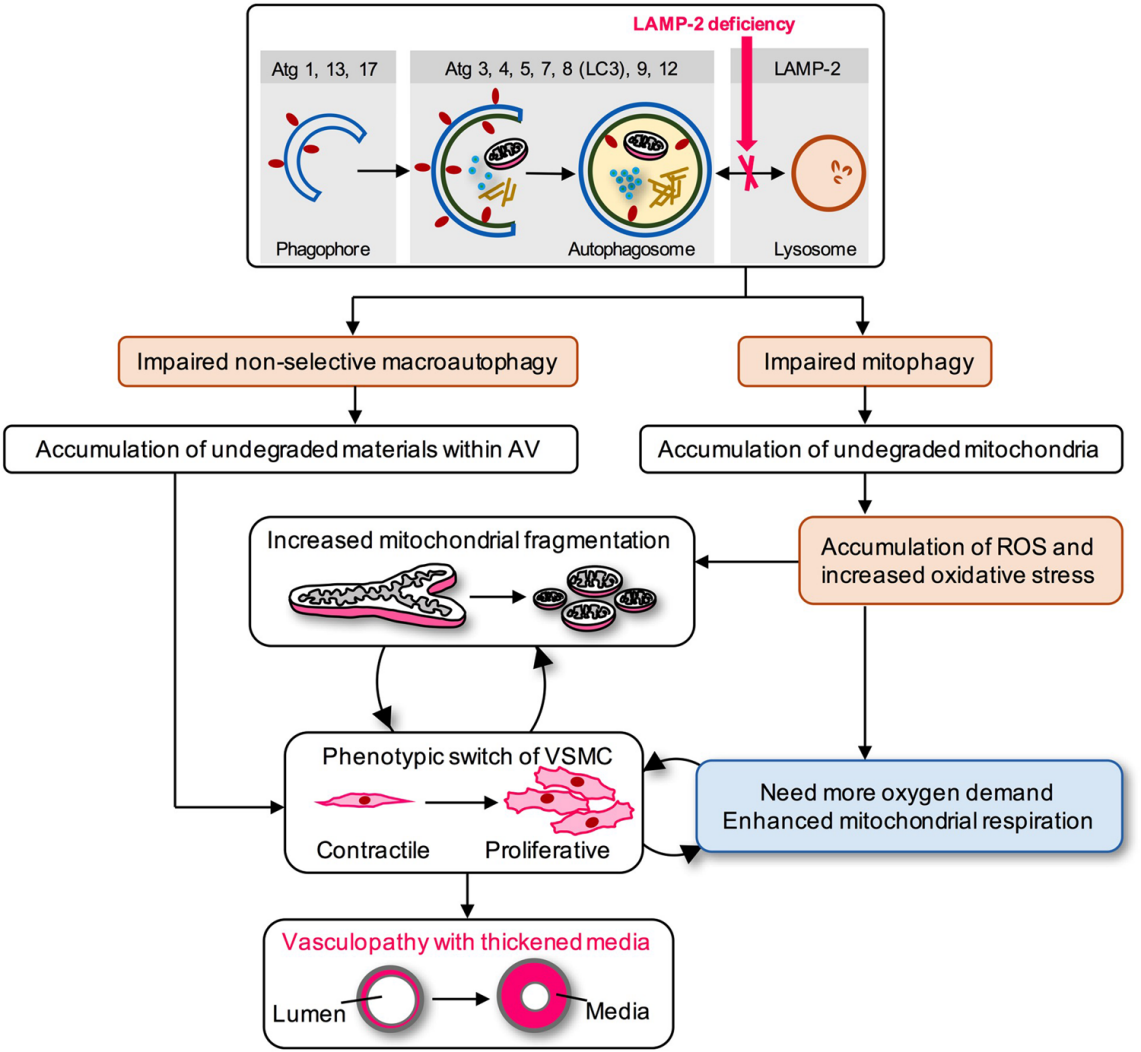
Hypothetical model of vasculopathy in Danon disease. LAMP-2 deficiency is a primary event that triggers serial pathologic cascades leading to vasculopathy. LAMP-2 protein mediates the final step of macroautophagy by facilitating the fusion between AV and lysosomes. Damaged mitochondria sequestrated by AV are subsequently targeted for lysosomal degradation via mitophagy. Mitochondria are known as a major source of ROS, a mediator of vasculopathy. Mitophagy plays a role in the clearance of ROS through digestion of damaged mitochondria. We found the accumulation of mitophagic vacuoles, together with non-selective AV, in the cytoplasm of VSMC that comprise the medial layer of muscular arteries. Based on the data, we propose that the following two events are important to develop ischemic vasculopathy: (i) excessive oxidative stress due to impaired mitophagy, and/or (ii) storage of toxic metabolites arising from defective, non-selective autophagic pathways. Mitochondrial fragmentation and augmented mitochondrial respiration observed in our study may result from an adaptive response to rapid proliferation of a synthetic phenotype and/or as a direct consequence of increased oxidative stress in VSMC.

Among various morphologic types of AV observed in VSMC under LAMP-2 deficiency, we particularly focused on mitophagy, which plays a key role in regulating VSMC proliferation through the maintenance of mitochondrial quality and/or the cell cycle^35,36^. Mitochondria are the principal source of ROS, which subsequently trigger various cytotoxic reactions. Our present results with LAMP-2 deficiency of humans, mice, and cultured VSMC suggest that impaired mitophagy could increase intracellular ROS and activate proliferation. This notion is supported by the following observations in our study: (1) accumulation of damaged and superfluous mitochondria in VSMC, (2) biochemical increase in ROS, (3) enhanced mitochondrial respiration, (4) active proliferation, and (5) histologically proven medial hypertrophy (Fig. 8). Consistent with our view, previous studies have shown that excessive ROS promote the phenotypic switch of VSMC from contractile to proliferative state^32,37,38^. Exposure of VSMC to platelet-derived growth factor (PDGF), a stimulant for VSMC proliferation, increased mitochondrial fragmentation^39^ as well as mitochondrial respiration^40^, with concomitant cell proliferation. Our data together with others support the notion that the proliferative phenotype likely represents a physiological adaptation to oxidative stress, so that the cells can ensure sufficient respiratory capacity.

In conclusion, the pathogenesis of vasculopathy is multifactorial and caused by complex mechanisms. Our study with LAMP-2–deficient patients and mice suggests that autophagy, particularly mitophagy, represents one of the important biological pathways to maintain arterial structural integrity through regulation of mitochondrial dynamics and function, which are critical determinants of the proliferation activity of VSMC. The maintenance of optimal autophagic activity should help prevent an unnecessary proliferative response of VSMC, thereby representing a critical therapeutic target in both the rare genetic form and the more common, age-related form of vasculopathy.

## Materials and Methods

### Clinical and genetic studies

#### *LAMP2* sequencing analysis

Genomic DNA was isolated from peripheral blood leukocytes using the QiaAmp DNA Blood kit. Nine exons and flanking intronic regions of the *LAMP-2* gene (NM_013995.2) were amplified with the primers reported previously^4^. The sequences of the amplicons were directly determined on both forward and reverse strands using the BigDye Terminator Cycle Sequencing Kit (PE Biosystems), and then samples were subjected to electrophoresis using an ABI 310 Genetic Analyzer (PE Biosystems). For human studies, informed consent for medical diagnosis and research was obtained from the patients and family. The human study protocols were approved by the ethical committee of research in Kansai Medical University and National Institute of Neuroscience, National Center of Neurology and Psychiatry. All experiments were performed in accordance with relevant guidelines and regulations.

#### Analysis of muscle biopsy specimens

A muscle biopsy was taken from the quadriceps femoris muscle in patient II-2 of family 1. All biopsy samples were frozen in liquid nitrogen-cooled isopentane for histochemistry and fixed in buffered glutaraldehyde for transmission electron microscopy (TEM). Ten-micrometer-thick transverse frozen sections were stained with hematoxylin and eosin (H&E) and subjected to a battery of histochemical methods. For TEM, biopsy specimens were fixed in buffered 2% glutaraldehyde at pH 7.4, post-fixed in osmium tetroxide and then embedded in epoxy resin. Ultra-thin sections were stained with uranyl acetate and lead citrate, and then examined with an H-7000 electron microscope (Hitachi, Tokyo, Japan). We performed Western analysis for skeletal muscles from the affected son and control subject as described^41^.

### Mouse studies

#### Preparation of mouse tissues

LAMP-2–deficient mice consisting of three genotypes (*Lamp2*^−/−^, *Lamp2*^y/−^ and *Lamp2*^+/−^) and the wild-type of the same strain C57BL/6 J (*Lamp2*^y/+^) were a gift from Professor Saftig (Department of Biochemistry at the Christian-Albrechts-Universität Kiel) and were maintained in the National Institute of Neuroscience, National Center of Neurology and Psychiatry, Tokyo, Japan (Supplementary Table S2). Mice were anesthetized, and perfused through the left ventricle with saline followed by 2% paraformaldehyde (PFA). The brain, heart, and femoral arteries were manually excised (Supplementary Fig. S13). The animal study protocols were approved by the ethical committee of research in the National Institute of Neuroscience, National Center of Neurology and Psychiatry. All experiments were performed in accordance with relevant guidelines and regulations of institute’s Animal Investigation Committee.

#### Histopathological analysis and morphometric assessment

The dissected specimens were immediately dehydrated by immersing them in a graded series of ethanol solutions. The dehydrated samples were embedded in paraffin, sectioned into five-micrometer-thickness sections, and stained with H&E and Masson trichrome. For morphometric assessment, a serial section from the entire femoral artery was investigated (Supplementary Fig. S14). Quantitative analyses were done using Image J software (NIH, Bethesda, MD).

#### Immunofluorescence experiments on frozen tissues

Ten-micrometer-thick cryosections of arteries, which were embedded in OCT compound, were prepared from the wild-type and *Lamp2* knockout mice; and then were stained for autophagosomes, α-smooth muscle actin (α-SMA), and vimentin. Cryosections were washed with PBS for 5 min and permeabilized with 0.25% Triton X-100 in PBS for 10 minutes. After washing with PBS, the sections were blocked in Blocking One Histo® (Nacalai Tesque, 06349-64) for 30 min at room temperature.

For the detection of autophagosomes in VSMC, Zenon rabbit IgG labeling kits (Molecular Probes, Z-25302 and Z-25307) were used according to the manufacturer’s instructions. The first mixture of antibodies was combined from rabbit monoclonal anti-LC3A/B (1:500, Cell Signaling, #12741) and goat anti-rabbit IgG Alexa Fluor 594 (1:500, Abcam, ab150088). The second mixture was combined from rabbit monoclonal anti-α smooth muscle actin (1:500, Abcam, ab32575) and goat anti-rabbit IgG Alexa Fluor 488 (1:500, Abcam, ab150085). Each mixture of antibodies was incubated in a humidified chamber for 60 min at room temperature.

For staining of α-SMA or vimentin, cryosections of femoral arteries were double stained with a mixture of two primary antibodies: goat polyclonal anti-α-SMA (1:500, Abcam, ab21027) or rabbit monoclonal anti-vimentin (D21H3) XP antibody (1:200, Cell Signaling, #5741), and rat monoclonal anti-CD31 antibody (1:500, GeneTex, GTX54379). This was followed by staining with a mixture of two corresponding secondary antibodies. Nuclei were counterstained using DAPI (200 ng/mL) for 10 minutes at room temperature in the dark. All images were acquired using identical gain settings on the ZEISS LSM 510 META Confocal Laser Scanning Microscope.

#### Quantitative analysis of corrected total tissue fluorescence (CTTF)

The quantitative analyses were performed as previously described with some modifications^42^. Four representative cross-sectional images of femoral arteries were chosen from each group (*Lamp2* knockout and wild-type mice) for further quantification. For each artery, five distinct segments from the vascular walls were randomly selected using ImageJ software, followed by the measurement of segmental area and integrated density. For each genotype, parameters from twenty areas were obtained (Supplementary Fig. S15). After that, a region without fluorescence next to a selected arterial segment was measured for “mean background fluorescence”. The following formula was used to calculate corrected total tissue fluorescence: CTTF = integrated density − (area of selected segment × mean background fluorescence).

#### Ultrastructural analysis of arteries

The dissected specimens were post-fixed with 2% glutaraldehyde overnight at 4° C. The specimens were trimmed and washed with PBS, incubated in phosphate-buffered 1% osmium tetroxide for 1 hour, dehydrated in ethanol, and embedded in resin. Ultra-thin sections were stained with uranyl acetate and lead citrate. The quantification of AV density was performed as described previously with some modifications^43^. Ten distinct 4.25-µm square sections under larger magnification (x47,000) were randomly chosen from different fields, and the sum of these was used to determine the total number of AV. Two independent investigators, who were blinded to the origin of mice, inspected the images.

### Cell culture

#### Cell culture and LAMP-2 small interfering RNA (siRNA) treatment

The human brain VSMC were obtained from ScienCell Research Laboratories (Carlsbad, CA, USA, #1100) and cultured using smooth muscle cell medium (#1101, ScienCell) consisting of 500 mL basal medium, 10 mL fetal bovine serum (#0010), 5 mL of smooth muscle cell growth supplement (#1152), and 5 mL of penicillin/streptomycin solution (#0503). All experiments were done on human brain VSMC that underwent four passages. Twenty-four hours before treatment, cells were seeded at a density of 2 × 10^4^ cells/ml on poly-L-lysine coverslips in a 12-well plate for immunocytochemistry or at a cell density of 5 × 10^4^ cells/ml in a 6-well plate for immunoblotting or flow cytometry.

Human brain VSMC were transfected with LAMP-2 siRNA (Santa Cruz, sc-29390) or scramble siRNA (Santa Cruz, sc-37007) (control) using X-tremeGene 9 DNA transfection Reagent (Roche, 06-365-787-001) with a 6:1 ratio, according to the manufacturer’s protocol. Transfected cells were incubated for 48 hours at 37 °C before further processing.

#### Immunocytochemistry for detection of autophagosomes, α-SMA, and vimentin

Human brain VSMC grown on coverslips were fixed in 4% PFA in PBS for 20 min at room temperature. After rinsing in PBS, cells were permeabilized with 0.1% Triton X-100 for 5 min, followed by blocking in 5% goat serum (Wako, 143-06561) for 30 min at room temperature. Autophagosomes, α-SMA, and vimentin were visualized by incubating cells for 60 min at room temperature with rabbit monoclonal anti-LC3A/B (1:500, Cell Signaling, #12741), rabbit monoclonal anti-α-SMA (1:200, Abcam, ab32575), and rabbit monoclonal anti-vimentin (D21H3) XP (1:200, Cell Signaling, #5741), respectively. In all immunocytochemisty experiments, cells were double-stained with mouse monoclonal anti-LAMP-2/CD107b antibody (1:200, Novus Biologicals, NBP2–22217) for estimation of LAMP-2 siRNA transfection efficiency. Nuclei were counterstained using DAPI and coverslips were mounted with Prolong Gold antifade reagent (Invitrogen, P36930).

#### Monitoring autophagic flux

To analyze autophagic flux, VSMC were transfected with scramble siRNA or LAMP-2 siRNA. The transfect cells were also labelled with the Premo autophagy tandem sensor RFP-GFP-LC3B (Invitrogen, P36239) on the same day, according to the manufacturer’s protocol. On the next day, the cells were either treated or not treated with chloroquine (60 µM, Invitrogen) and incubated for 24 hours. Cells were then fixed with 4% PFA and probed with mouse monoclonal anti-LAMP-2 antibody. In this assay, the autophagosomes are positive for both green (GFP) and red (RFP), which generate yellow puncta. Once autophagosomal-lysosomal fusion occurs, GFP is quenched under acidic conditions within lysosomes, resulting in red-colored autolysosomes. The numbers of autophagosomes as well as autolysosomes were quantified for 20 cells from each group.

#### Determination of apoptosis

Nuclei of 4% PFA-fixed cells were counterstained using Hoechst 33342 (10 µg/mL, Invitrogen, H3570). LAMP-2 protein and actin filaments were labeled with mouse monoclonal anti-LAMP-2 antibody and Phalloidin (1:50, Invitrogen, A22284), respectively. Nuclear morphologic changes that are characteristic of apoptosis, such as condensation and fragmentation, were used as a marker. Representative cells (*n* = 200) from the control and LAMP-2 siRNA-treated groups were randomly chosen for quantification.

#### Determination of proliferation

Detached cells were scraped off with PBS, collected by centrifugation at 200 × G for 3 min. Cells (1 × 10^6^) were suspended in 100 µL staining buffer (BD Biosciences, 563794) and incubated with Fc block (BD Biosciences, 564220) for 10 min at 4 °C, then stained with anti-Human Ki67 (Affymetrix eBioscience, 25-5699-42) for 15 min. Cells were subsequently fixed in fixative solution IOTest 3 (Beckman Coulter, A07800) for 10 min and then washed twice with PBS. Cells were finally suspended in 500 µL sheath buffer and analyzed by BD FACSCANTO II (Biosciences, USA). A forward scatter (FSC) and side scatter (SSC) were used to select the whole population. The Ki67-positive cells were gated to calculate mean fluorescence intensity (MFI). The samples were run on a flow cytometer, collecting at least 20,000 events for each assay. Data analysis was conducted by FlowJo software (version 7.6.5, FlowJo LLC, Ashland, Oregon) (Supplementary Fig. S11).

#### Mitochondrial dynamics analysis

After 48 hours of transfection, the growth media was discarded and cells were labeled with pre-warmed staining solution containing MitoTracker Red CMXRos (100 nM, Invitrogen, M7512). After incubation for 20 min at 37 °C, cells were fixed with 4% PFA, permeabilized with 0.1% Triton X-100, and blocked in 5% goat serum before probing with mouse monoclonal anti-LAMP-2 antibody. The quantification of mitochondrial morphology was performed as previously described with some modifications^44^. Twenty representative cells from each group, control and LAMP-2 siRNA-treated cells, were randomly chosen for quantification. Within cells, three cytoplasmic regions of interest (ROI) of 10 × 10 µm area were defined, excluding condensed perinuclear mitochondria. For each ROI, the number of mitochondria and area of individual mitochondria were measured by ImageJ (Supplementary Fig. S16). In this study, a decrease in mitochondrial individual area and conversely an increase in the number of mitochondria were used to define fragmentation.

To examine the colocalization of DRP-1 with mitochondria, after preloading with MitoTracker Red CMXRos, cells were sequentially probed with rabbit monoclonal anti-DRP1 antibody (1:250, Abcam, ab184247) and then mouse monoclonal anti-LAMP-2 antibody. Each first antibody was followed by a corresponding secondary antibody. Colocalization between MitoTracker and DRP-1 was quantified using ZEISS LSM 510 META software, and 20 cells from each group, control and LAMP-2 siRNA-treated cells, were analyzed. The peripheral cytoplasmic region was selected to quantitate the extent of colocalization (Supplementary Fig. S17).

To analyze mitochondrial dynamics in live cells, VSMC were plated on 35-mm glass-bottom dishes (Matsunami, D110310). After 24 hours of transfection, cells were labelled with CellLight GFP-Tubulin (Invitrogen, C10509) for indirect estimation of LAMP-2 siRNA transfection efficiency (Supplementary Fig. S21). On the day of the experiment, cells were loaded with MitoTracker Red CMXRos for 20 min and were recovered in 37 °C growth media. Cells were imaged live by a ZEISS LSM 510 META microscope equipped with a 63× oil-immersion objective as previously described with some modifications^45^. The cells were maintained at 37 °C under 5% CO_2_ during 2 hours of investigation. Images were acquired in Z stacks of 6 planes at 0.4-μm intervals every 10 seconds over a period of 3 minutes for each cell and processed by ZEISS LSM 510 META software.

#### Mitochondrial respiration analysis

The oxygen consumption rate (OCR) was measured in intact human brain VSMC using the Seahorse XFp analyzer (Agilent Technologies, Santa Clara, CA, USA). Cells (4 × 10^4^ cells per well) were seeded into 6 wells of an 8-well XFp Miniplate. After 24 hours of seeding, transfection of LAMP-2 siRNA was performed in 3 wells, while the other 3 wells were not transfected for use as a control. Cells were then incubated for 48 hours before the mitochondrial assay. On the day of assay, cells were washed and replaced with 180 µL/well of XF Base Medium (#103335–100) supplemented with 25 mM D-glucose, 1 mM Pyruvate, 2 mM L-glutamine, and adjusted to a pH of 7.4. The OCR measurement was performed after equilibrating the cells in the XF assay shereicmedia for 30 min. Oligomycin (1 µM), carbonyl cyanide 4-(trifluoromethoxy) phenylhydrazone (FCCP; 1 µM), and rotenone + antimycin A (0.5 µM each) were serially injected, and the OCR was recorded following the manufacturer’s protocol for Cell Mito Stress Test. Data were normalized to total protein in each well. For normalization, cells were lysed using 20 μl/well of protein lysis buffer. The total protein in each well was determined by DC Protein Assay (Bio-Rad, #500–0116). Data analysis was conducted by Wave software (version 2.4.0, Agilent Technologies).

#### Assessment of H_2_O_2_

We used the dichlorofluorescein (DCF) assay to measure total intracellular H_2_O_2_ under adhesive and non-adhesive conditions. On the day of the experiment, cells were loaded in the presence or absence of 2.5 µM 2′,7′-dichlorodihydrofluorescein diacetate (H_2_DCF-DA) (Invitrogen, D399) for 30 min, and the cells were recovered with growth media for 30 min at 37 °C. For the solid-phase assay, adherent cells were fixed with 4% PFA and counterstained with DAPI. For the liquid-phase assay by flow cytometry, cells were rinsed once with PBS. Detached cells were scraped off with freshly new PBS, followed by centrifugation at 200 × G for 3 min. Cellular pellets were dissolved in PBS and briefly vortexed before the analysis. The dye was detected by BD FACSCANTO II (Biosciences, USA) equipped with a 488-nm argon-ion laser. First, the fluorescence of untreated dye-unloaded cells was examined to set the scatter signals and the background fluorescence. Next, control cells and LAMP-2 siRNA-treated dye-loaded cells were run on a flow cytometer, collecting at least 20,000 events for each assay. The MFI of the whole cell population was calculated (Supplementary Fig. S12).

#### Western analysis

Cells were lysed using buffer consisting of 1% Triton X-100, 0.1% sodium dodecyl sulfate (SDS), 150 mM NaCl, 50 mM Tris-HCl, 5 mM Ethylenediaminetetraacetic acid (EDTA), and proteinase inhibitor cocktail (Roche, 04-693-124-001). The protein concentration was determined by DC Protein Assay. Equal amounts of lysates were separated by SDS-PAGE and transferred to PVDF membranes (GE Healthcare, 10600100) using a semidry blotting system in transfer buffer (25 mM Tris base, 190 mM glycine, 20% methanol). The membranes were subsequently blocked with 2% ECL Prime blocking agent (GE Healthcare, RPN418V) and incubated with primary antibodies, followed by corresponding ECL peroxidase labelled secondary antibodies (1:10,000, GE Healthcare). Immunoreactivity was detected by the ECL reaction (GE Healthcare, RPN2235). The blots were exposed to ECL films (GE Healthcare) before they were visualized by scanning densitometry. After detection of the protein of interest, to control for equal loading, blots were submerged in stripping buffer (62.5 mM Tris-HCl pH 6.7, 100 mM 2-mercaptoethanol, and 2% SDS) for 30 min at 50 °C before blocking, and then reprobed with mouse monoclonal anti-GAPDH antibody (1:1000, Wako, 014-25524). Quantitative measurement of bands were obtained by Image J software and expressed as a ratio of the amount of protein of interest relative to GAPDH.

#### Statistics analysis

Statistical analyses were performed using IBM SPSS Statistics version 21. Graphs were designed using GraphPad Prism version 7.0. Quantitative values are represented as the mean ± SD. All data in this study fit a normal distribution. The numbers of mice analyzed are indicated *(n)* in the figure legends. All cell experiments were performed at least three times. Vascular morphometric parameters of mice were analyzed by two-tailed *one-way ANOVA* followed by *Tukey*’s post-hoc test. Direct comparisons between two groups were performed using two-tailed *Student’s* t-tests.

## Electronic supplementary material

Supplementary information
