# Supplementary material for: Small-Vessel Vasculopathy Due to Aberrant Autophagy in LAMP-2 Deficiency
Source: Sci Rep. 2018 Feb 20;8:3326. doi: 10.1038/s41598-018-21602-8 (PMC5820257; doi:10.1038/s41598-018-21602-8)
Supplement: Supplementary file 1 — Supplementary information [file 41598_2018_21602_MOESM1_ESM.pdf]

SREP-17-28990B

Supplementary Information

Small-Vessel Vasculopathy Due to Aberrant Autophagy in LAMP-2 Deficiency

Huan T. Nguyen, Satoru Noguchi, Kazuma Sugie, Yoshiyuki Matsuo, Chuyen T.H. Nguyen, Hitoshi Koito, Ichiro Shiojima, Ichizo Nishino, Hiroyasu Tsukaguchi

Table of contents for supplementary information

Supplementary items	Descriptive Title	Page
**Figure S1**	Brain MRA and MRI scan in affected patients with *LAMP2* mutation.	3
**Figure S2**	Post-bypass surgery MRA of the mother with heterozygous *LAMP2* mutation.	4
**Figure S3**	Single-photon emission computed tomography scan of the mother with heterozygous *LAMP2* mutation at the age of 50 years.	4
**Figure S4**	Computed tomography angiography (CTA) scan of the mother with heterozygous *LAMP2* mutation at the age of 50 years.	5
**Figure S5**	Cardiac phenotypes of the heterozygous mother and hemizygous son with *LAMP2* mutation.	6
**Figure S6**	Histochemistry and electron micrograph of biopsied skeletal muscle of affected boy.	7
**Figure S7**	Gene structure, membrane topology model of human *LAMP2* gene, and location of the mutations.	8
**Figure S8**	Role of LAMP-2 in autophagy.	9
**Figure S9**	Distinctive effects of LAMP-2–deficiency on three types of muscle tissues.	10

Table of contents for supplementary information *(continuous)*

Supplementary items	Descriptive Title	Page
**Figure S10**	Phenotypic changes in cultured human brain VSMC.	11
**Figure S11**	Flow cytometric analysis for quantification of Ki67 in human brain VSMC.	12
**Figure S12**	Flow cytometric analysis for quantification of DCF in human brain VSMC.	13
**Figure S13**	Dissection methods for mouse tissues.	14
**Figure S14**	Morphometric assessment of femoral arteries.	14
**Figure S15**	Quantitative analysis of corrected total tissue fluorescence (CTTF).	14
**Figure S16**	Analysis of mitochondrial dynamics.	15
**Figure S17**	Analysis of DRP-1 recruitment to mitochondria.	15
**Figure S18**	Full unedited gel for Figure 1e.	16
**Figure S19**	Full unedited gel for Figure 4e.	17
**Figure S20**	Full unedited gel for Figure 5c.	18
**Figure S21**	Decreased expression of tubulin in LAMP-2–deficient human brain VSMC.	19
**Table S1**	Clinical features and laboratory findings in two patients of the first family with *LAMP2* mutation.	20
**Table S2**	Genotypes and frequency of vasculopathy in muscular arteries of LAMP-2–deficient mice.	22
**Table S3**	General physical features and morphometric analysis of femoral arteries of LAMP-2–deficient mice.	23
**Table S4**	Comparison between female wild-type $Lamp2^{+/+}$ and female heterozygous $Lamp2^{+/-}$ mice.	24
**Table S5**	List of first antibodies and corresponding second antibodies.	25
**References**		26

Supplementary Figures

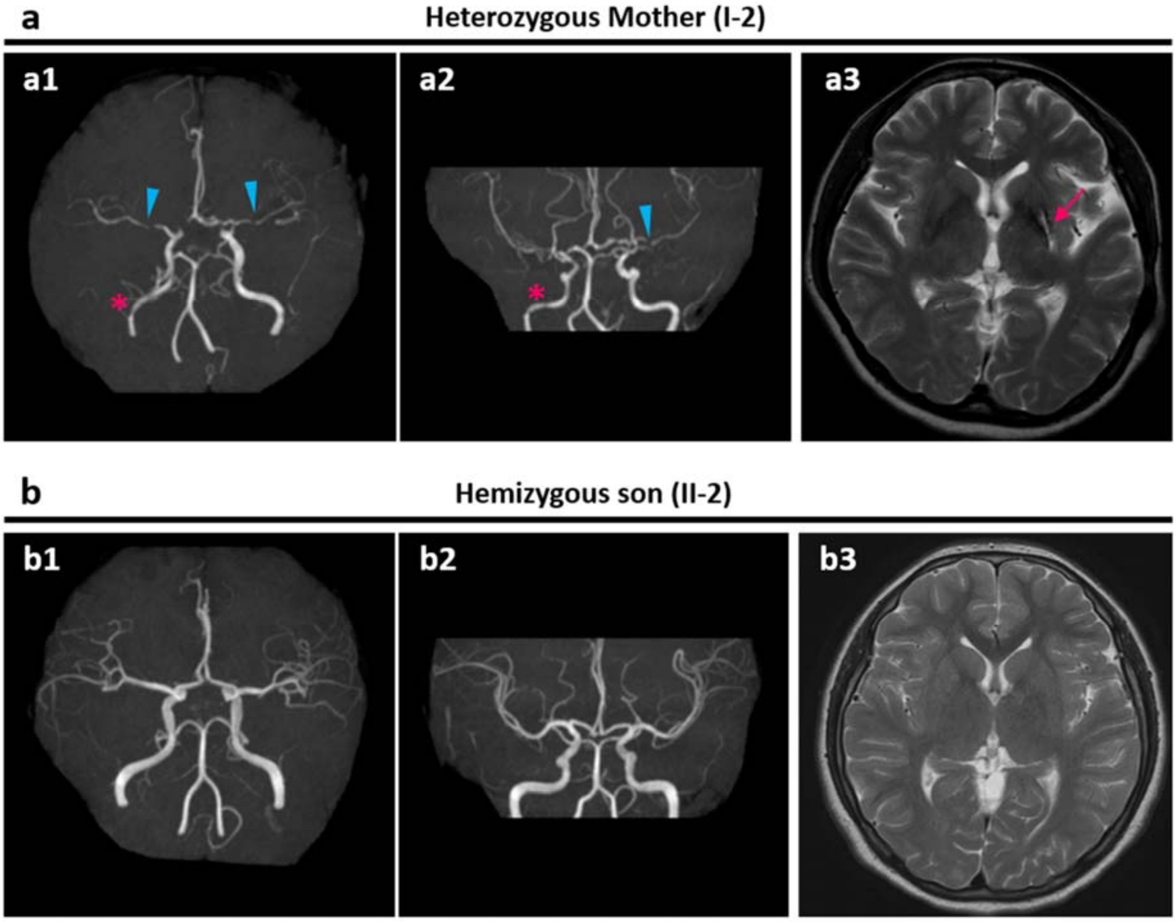

Supplementary Figure S1. Brain MRA and MRI scan in affected patients with *LAMP2* mutation. MRA (a1, b1: axial views; a2, b2: coronal views) and MRI images (a3, b3: axial views) of mother (heterozygous carrier of truncating *LAMP2* mutation) **(a)** and her son (hemizygote for the truncating mutation) **(b)** are shown. **(a1, a2)** The mother at age of 47 years exhibited a bilateral diffuse narrowing in the middle cerebral arteries (MCA, M2 portion) *(arrowheads)*. There was also mild, irregular stenosis in the proximal portion of right internal carotid artery (*asterisk*). **(b1, b2)** In contrast, the corresponding vasculatures of her son were intact at age of 16 years. **(a3)** A T2-weighted MRI scan of the mother at age of 50 years revealed a high intensity lesion in the left subcortical area surrounding the putamen, the region where is normally supplied by the MCA, indicating that brain parenchymal ischemia arose from the MCA stenosis *(arrow).* **(b3)** The son at age of 16 years did not have any morphologic abnormalities in the cortical development and structure-organization, nor ischemic changes.

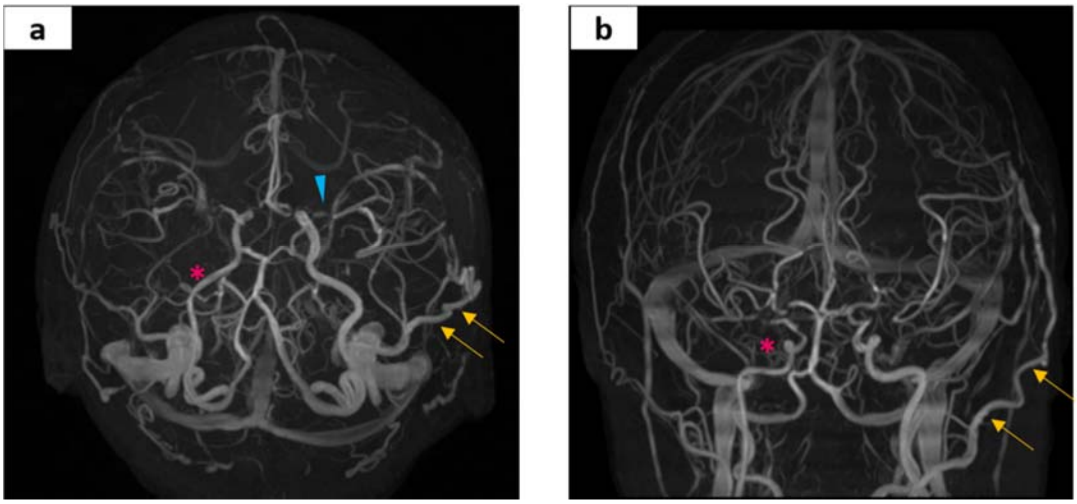

Supplementary Figure S2. Post-bypass surgery MRA of the mother with heterozygous *LAMP2* mutation. (a) axial, **(b)** coronal views of brain MRA images of the mother at the age of 50 years, who underwent the cerebral by-pass surgery at age of 47, are shown. The blood flow of the left middle cerebral artery (M2 portion, *arrowhead*) was restored by by-pass surgery, which connects the distal middle cerebral artery with the superficial temporal artery *(arrows)*. A slight irregularity of the right internal carotid artery (*asterisks*) is found.

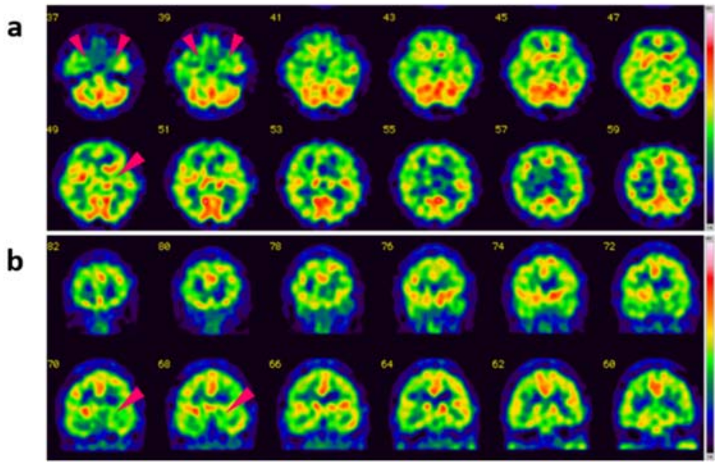

Supplementary Figure S3. Single-photon emission computed tomography scan of the mother with heterozygous *LAMP2* mutation at the age of 50 years. Functioning blood flow is evaluated by CT scan after intra-venous administration of Tc-99m Bicisate. It allows an approximate quantification of the cerebral blood perfusion, which is visualized by a colour spectrum of ranging from red *(hyper-perfused)* to blue *(hypo-perfused)* as indicated by the side standard bar. **(a)** Axial views show a reduced perfusion bilaterally in the region supplied by the anterior cerebral arteries *(arrowheads)*. **(b)** In coronal views, the middle cerebral artery region is also hypo-perfused particularly on the left side *(arrowheads)*.

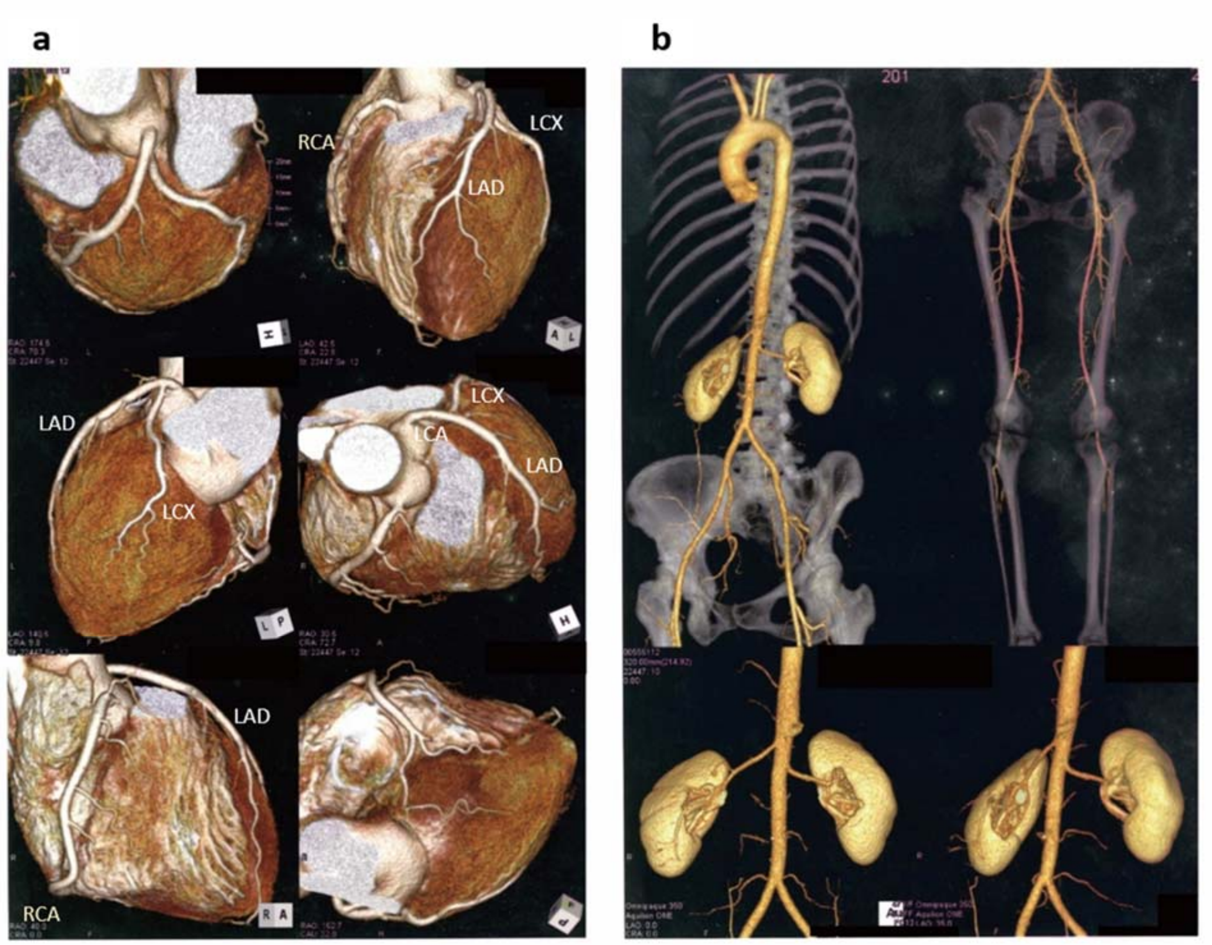

Supplementary Figure S4. Computed tomography angiography (CTA) scan of the mother with heterozygous *LAMP2* mutation at the age of 50 years. No significant sclerotic changes were found in coronary arteries **(a)**, as well as the main trunk of aorta and its peripheral branches **(b)**. The branches of coronary arteries refer to the left coronary artery (LCA), left circumflex artery (LCX), left anterior descending artery (LAD), and right coronary artery (RCA).

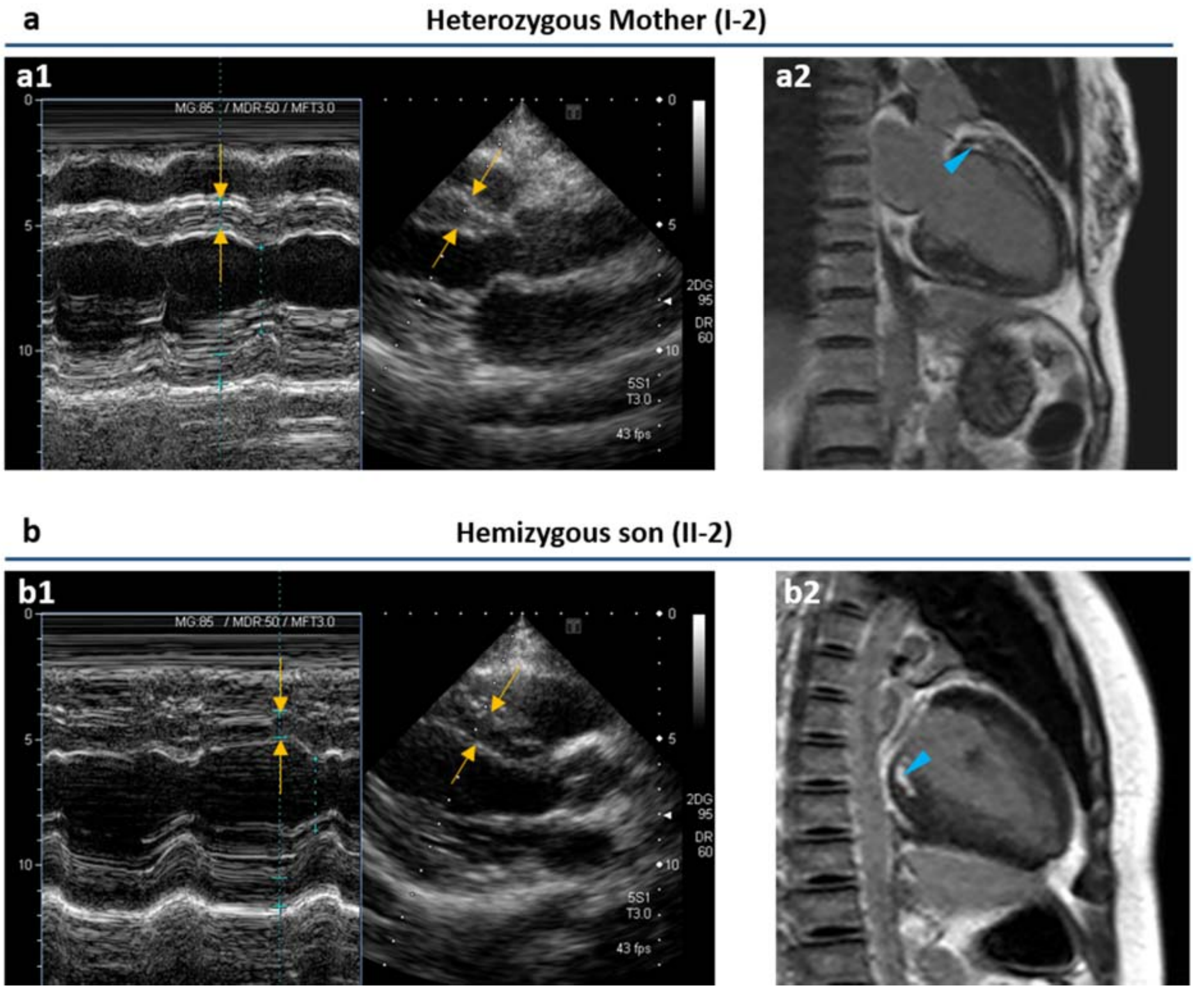

Supplementary Figure S5. Cardiac phenotypes of the heterozygous mother and hemizygous son with *LAMP2* mutation. (a) Images of mother at age of 50 years and **(b)** her son at age of 16 years are shown. Echocardiography revealed a mild concentric left ventricular hypertrophy in the mother **(a1)** as well as her son **(b1)**. The interventricular septum is thickened (11.8 mm, and 11 mm, for mother and son, respectively) at diastolic phase *(arrows)*. Cardiac magnetic resonance (CMR) images revealed an increase in left ventricular mass for both mother **(a2)** and son **(b2)**: 158 g and 219 g, respectively. Gadolinium administration study revealed the focal positive-late enhancement in the anterior (mother) and posterior (son) ventricular walls, indicating the focal myocardial fibrosis had developed from different myocardium locations *(arrowheads)*.

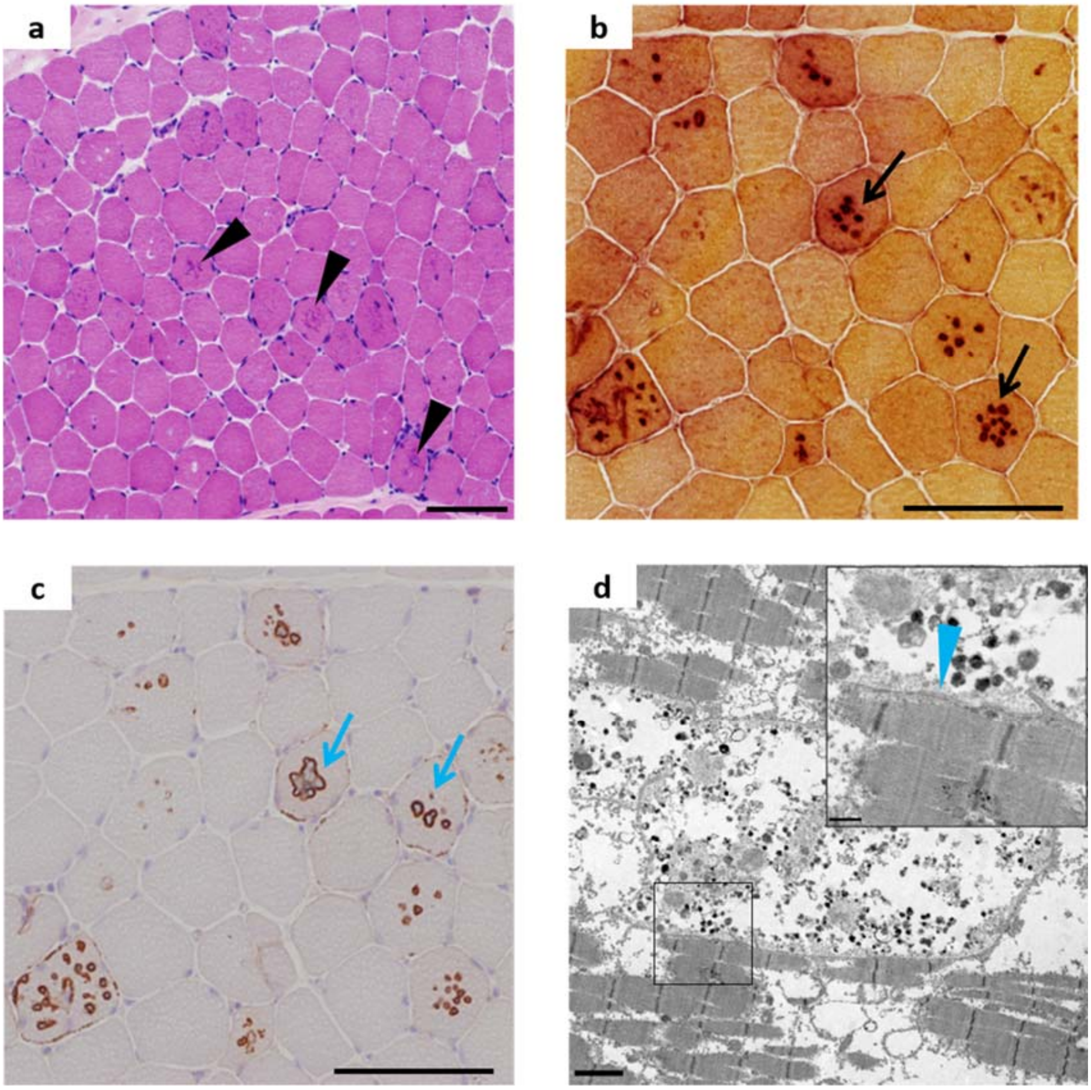

Supplementary Figure S6. Histochemistry and electron micrograph of biopsied skeletal muscle of affected boy. The biopsies were performed from femoral muscle in hemizygous male patient (II-2) with truncating *LAMP2* mutation at the age of 16 years. Light micrograph of skeletal muscle tissues from proband boy are shown **(a-c)**. **(a)** In hematoxylin & eosin stain, the basophilic, granular structures were seen in the cytoplasm of some myofibrils (*arrowheads*). There is mild variation in size of myofibers without necrotic or regenerative changes. These intracytoplasmic materials were positive for the nonspecific esterase *(black arrows)* **(b)** and acetylcholine-esterase *(blue arrows)* **(c)**. These pathologic features are consistent with those of autophagic vacuoles with unique sarcolemmal features (AVSF) reported for Danon disease[1]. Scale bars: (a, b, c) 100 µm. **(D)** Ultrastructural analysis showed AV among myofibrils. The larger magnification revealed the double-membrane *(arrowhead)*, indicating the vacuolar membrane has sarcolemmal features. Scale bars: (d) 2 µm; inset 0.5 µm.

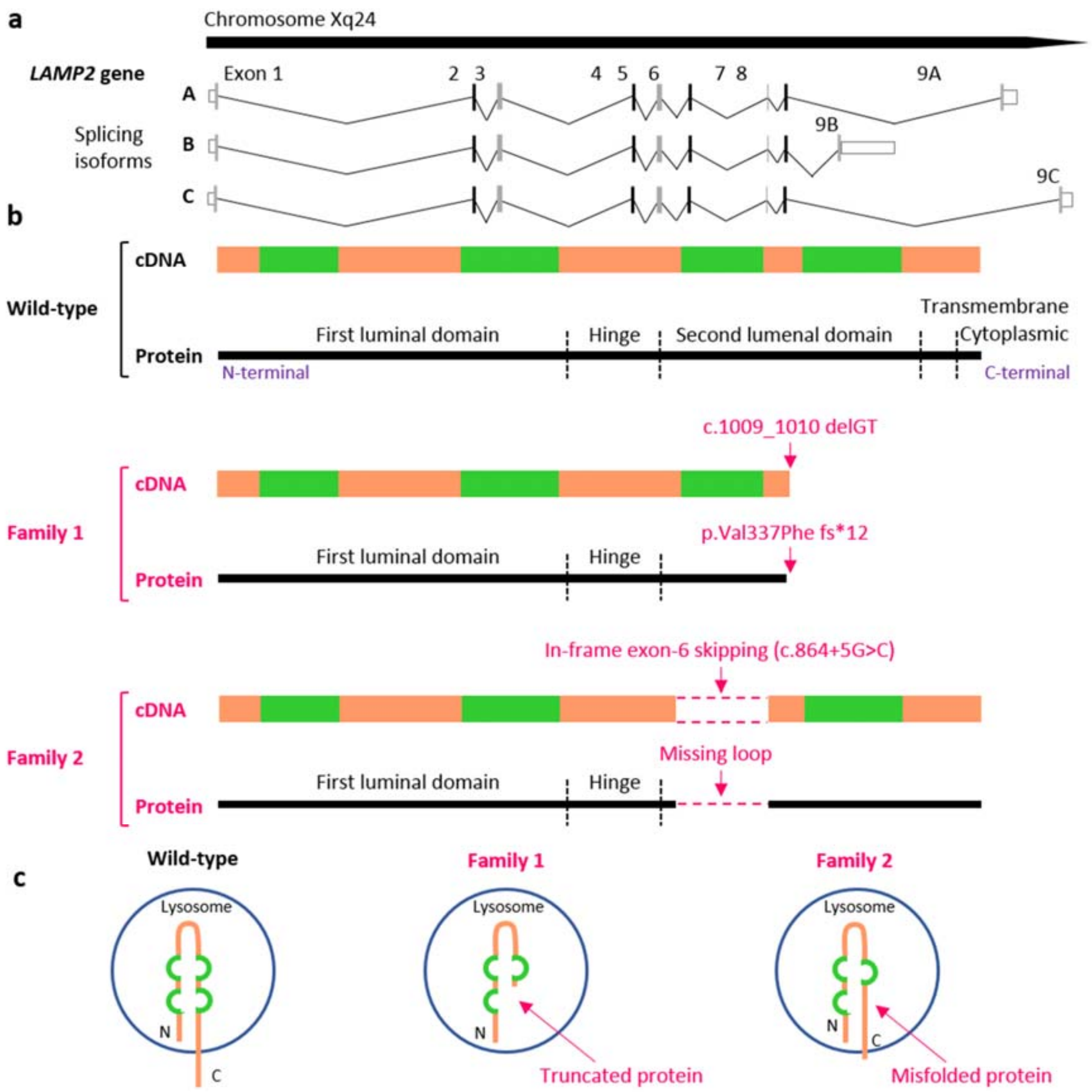

Supplementary Figure S7. Gene structure, membrane topology model of human *LAMP2* gene, and location of the mutations. (a) Splicing variants of *LAMP2*. Human *LAMP2* gene consists of total 9 exons. There are three distinct exon 9 in human, producing three alternative splicing isoforms: LAMP-2A, LAMP-2B, and LAMP-2C[2]. **(b)** Domain structures of LAMP-2 and effects of the mutations. Each isoform encodes three different transmembrane and cytoplasmic domains. *Green boxes* represent the four loop regions, each of which are internally formed by two flanking cysteine residues. In family 1, a frameshift mutation (c.1009_1010delGT) deletes the C-terminal one-third portions, which serves as a lysosome-targeting signal. In family 2, a splice-donor site mutation (c.864+5G>C, in intron 6) causes an in-frame exon-6 skipping, which potentially disrupts the intermolecular loop. **(c)** Membrane topology of wild-type and mutant LAMP-2 proteins. These disease variants are predicted to lose a proper membrane anchoring (family 1) or protein folding (family 2), thereby causing a functional LAMP-2 deficiency.

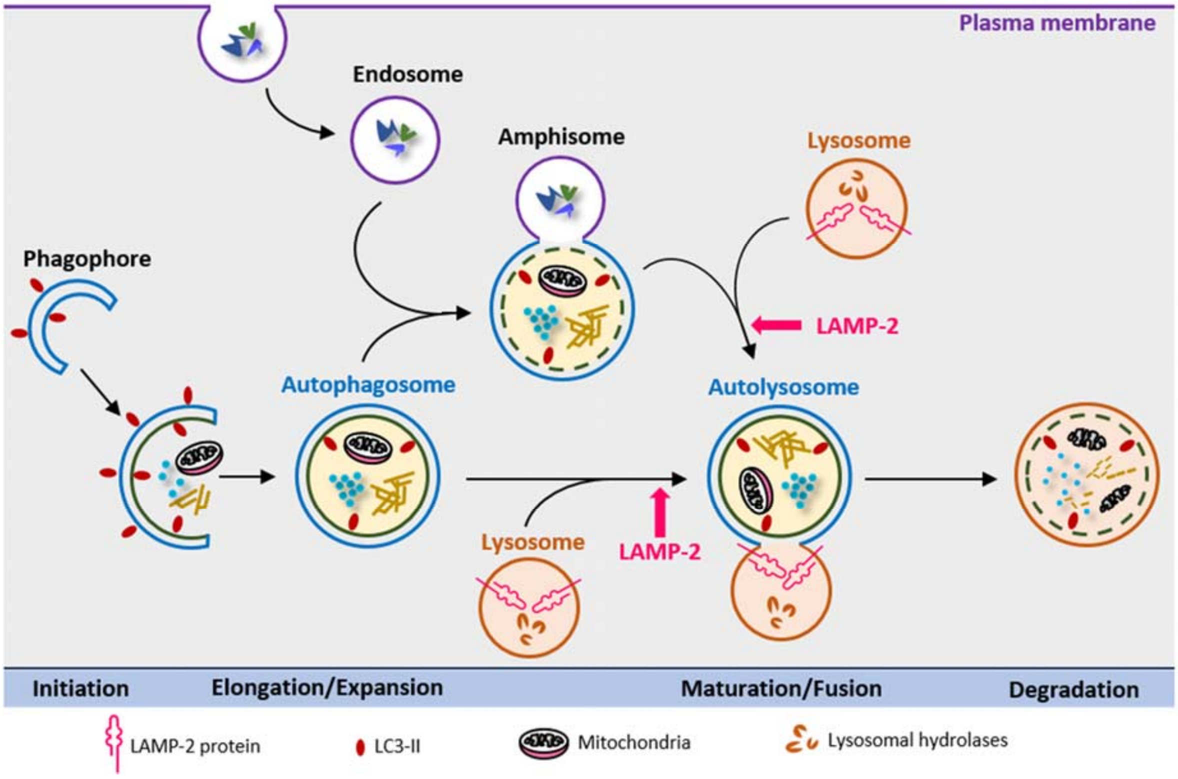

Supplementary Figure S8. Role of LAMP-2 in autophagy. Autophagy, a well-conserved, inducible process for bulk clearance of cytosolic components and organelles by delivering the metabolic wastes and organelles into lysosomes, is initiated by the formation of the phagophore and followed by a series of steps. Amphisome, represents AV of external origin, was formed by fusion of an autophagosome and endosome. Autolysosome, a product of fusion between an autophagosomes or an amphisome and lysosome, is facilitated by LAMP-2 protein *(red arrows)*. The defects in LAMP-2 protein are therefore expected to cause the cytoplasmic accumulation of AV, most of which likely proceed towards late phase of this fusion process. LAMP-2, lysosomal associated membrane protein 2; LC3, microtubule-associated protein 1 light chain. This figure was reproduced with permission of the copyright owner[3].

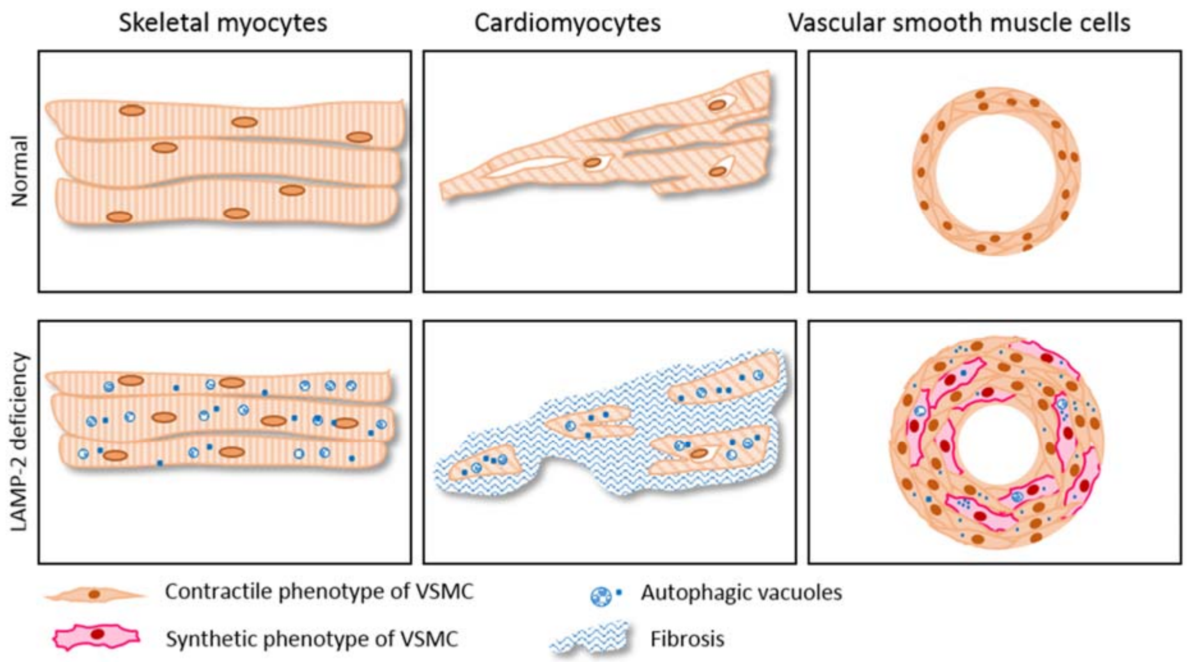

Supplementary Figure S9. Distinctive effects of LAMP-2–deficiency on three types of muscle tissues. There are three distinct muscle-cell types including cardiomyocyte, skeletal myocyte, and VSMC. Given that LAMP-2 plays a key role in macroautophagy, it is theoretically feasible that all three muscle-cell lineages may accumulate cytoplasmic autophagic vacuoles under the LAMP-2 deficiency. In Danon disease, myopathy and cardiomyopathy are constant features but the phenotype of VSMC remains elusive. Generally, skeletal muscles in Danon patients show mild to moderate degeneration which is characterized by size variability of myofibers and the absence of necrotic or regenerative changes[1]. In contract, cardiac muscles suffer from a prominent myofibrillar disruption, including substantial myocyte disarray, and accumulation of vesicles with fibrosis[4]. The two cell types, skeletal myocyte and cardiomyocyte are post-mitotic cells that stably remain in the G1 phase, thereby maintaining their terminally differentiated cell-types. In contrast, VSMC has a mitotic activity so that it can reversibly convert the phenotypes between the contractile and synthetic ones. The distinctive responses to LAMP-2 deficiency in these muscular tissues may also reflect that (a) relative LAMP isoform (LAMP-1 vs LAMP-2) as well as lysosomal hydrases[5], (b) skewed inactivation of *LAMP2* gene in female[6], (c) susceptibility to age-dependent decline in degradation pathways[7], (d) compositions of cytoskeletal elements and the extracellular matrix surrounding the myocytes[8].

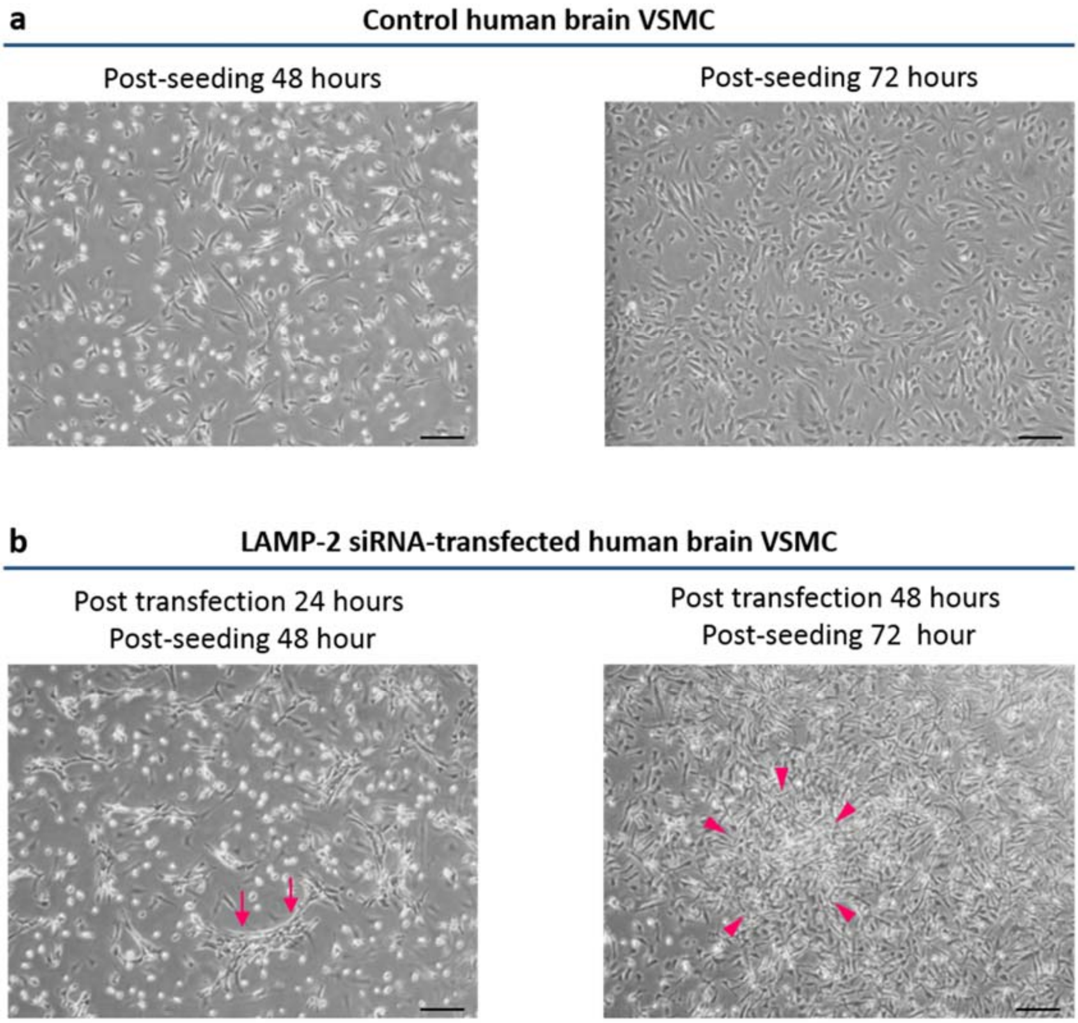

Supplementary Figure S10. Phenotypic changes in cultured human brain VSMC. Cellular morphology and growth were compared between control **(A)** and LAMP-2 siRNA-treated **(B)** human brain VSMC at the same fourth passage from primary isolation. Twenty-four hours prior to transfection, cells were seeded onto poly-L-lysine coverslips in a 12-well plate with an equal density for each well. A phase contrasted, live cell images were captured by the inverted light microscopy. Twenty-four hours after LAMP-2 siRNA transfection, most cells still grew separate each other evenly over the bottom, while some made the clumps *(arrows)*. Forty-eight hours after transfection, the LAMP-2 silencing cells tended to have epithelioid-shaped, thereby constituting a typical hill-and-valley pattern *(arrowheads).* Control cells spread out evenly over the plate with spindle-shaped morphology. The observation suggested that LAMP-2 deficiency accelerates a proliferation rate of VSMC and permits a faster acquisition of confluent state. Scale bar: 200 µm.

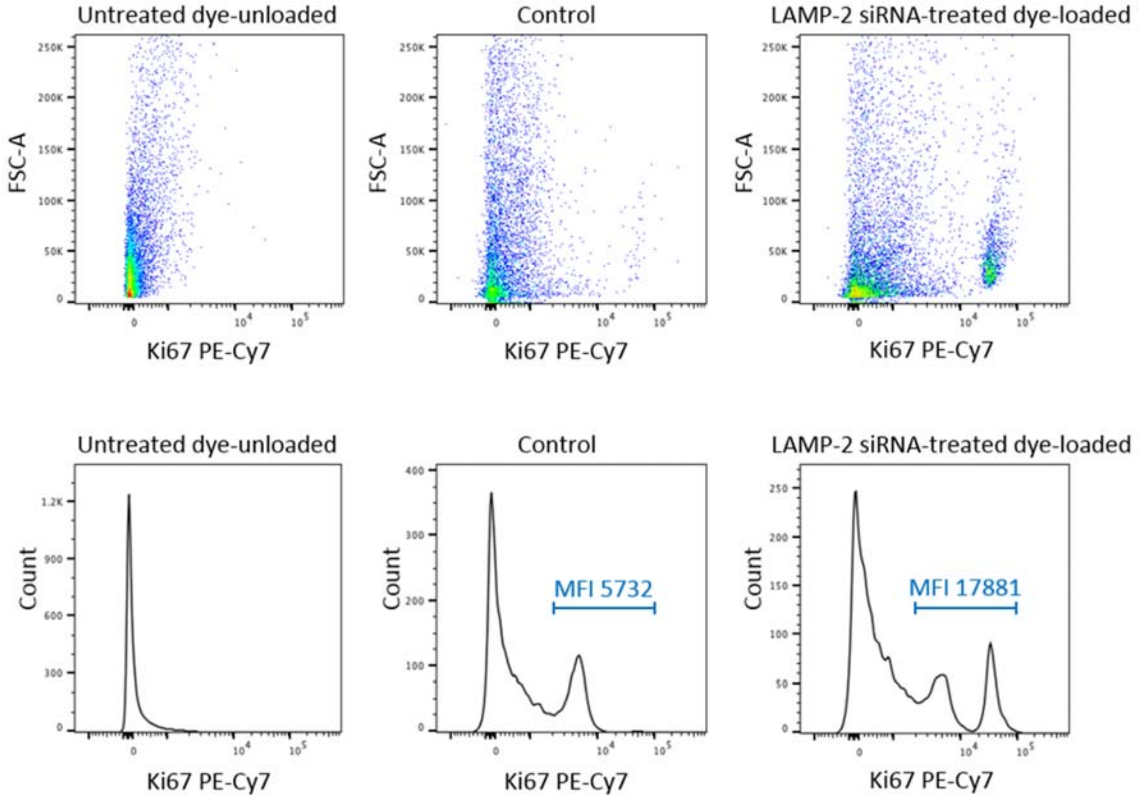

Supplementary Figure S11. Flow cytometric analysis for quantification of Ki67 in human brain VSMC. A representative experiment is shown by dot plot graphs (*upper panels*) and also by consistent histograms (*lower panels*). The expression of Ki67, a maker of cell proliferation, was measured in VSCM cell suspension by using BD FACSCANTO II (Biosciences, USA). A blank sample (that with no dyes nor treatment) was first analyzed in order to set as the scatter signals and the background fluorescence. Next, scramble siRNA-treated dye-loaded (control) and LAMP-2 siRNA-treated dye-loaded cells were run on a flow cytometer, collecting at least 20,000 events for each assay. Mean fluorescence intensity (MFI) was calculated from gated Ki67-positive cells. FSC-A, forward scatter pulse area.

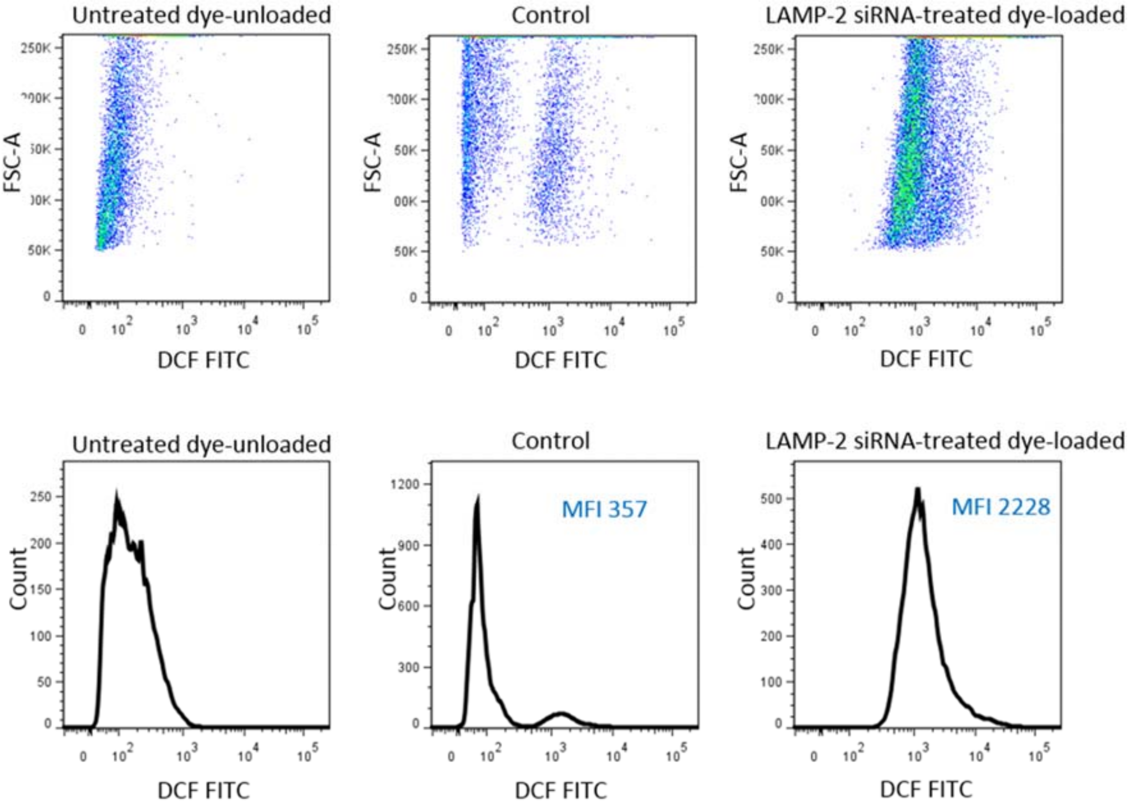

Supplementary Figure S12. Flow cytometric analysis for quantification of DCF in human brain VSMC. A representative experiment is shown by dot plot graphs (*upper panels*) and also by consistent histograms (*lower panels*). Fluorescence signals of dichlorofluorescein (DCF), an indicator of ROS activity, are measured in VSCM cell suspension by the flow cytometry BD FACSCANTO II (Biosciences, USA). The blank sample (that with no dyes nor treatment) was first analyzed in order to set as the scatter signals and the background fluorescence. Next, scramble siRNA-treated dye-loaded (control) and LAMP-2 siRNA-treated dye-loaded cells were run on a flow cytometer, collecting at least 20,000 events for each assay. In DCF assay, MFI was calculated from each whole cell population. FSC-A, forward scatter pulse area.

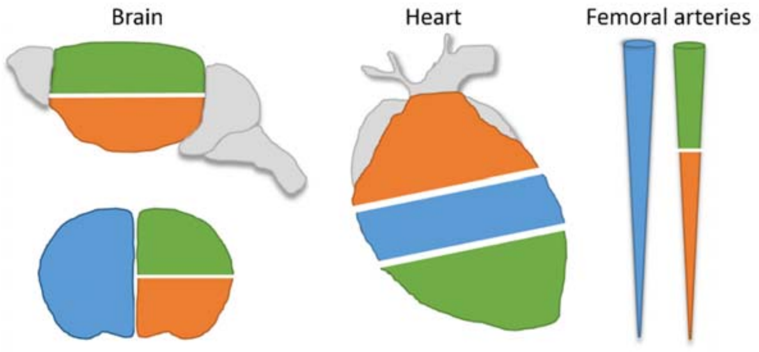

Supplementary Figure S13. Dissection methods for mouse tissues. The parts of tissues of interest were removed and dissected into distinct parts for histochemistry *(blue)*, immunofluorescence *(green or orange)*, and electron microscopy *(green or orange)*.

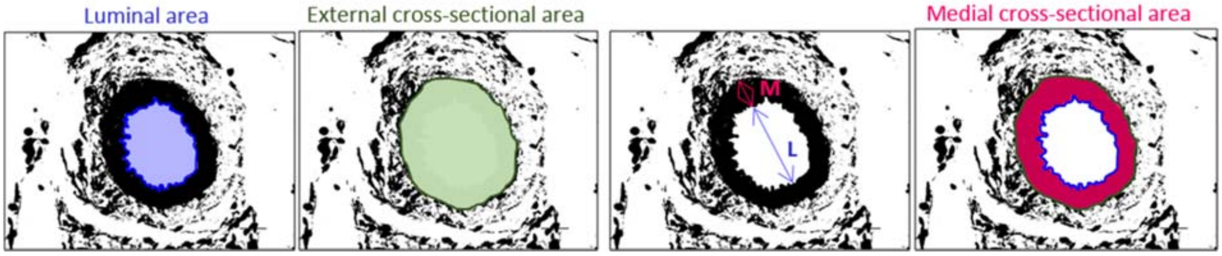

Supplementary Figure S14. Morphometric assessment of femoral arteries. Because of uniquely entire excision, only femoral arteries were able to be processed for the quantitation. Luminal area was detected using "tracing tool" of Image J. External cross-sectional area (external CSA) and media thickness (M) were manually measured. Medial cross-sectional area (CSA) was estimated by subtracting the luminal area from external CSA. Lumen diameter (L) was calculated from luminal area.

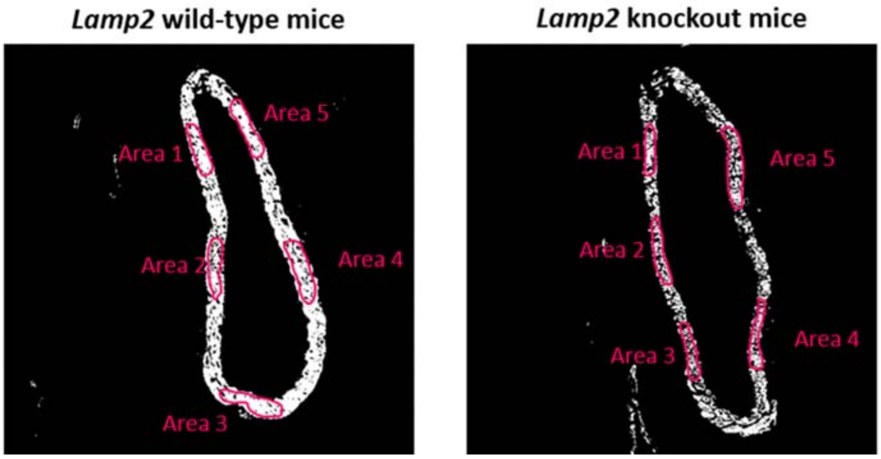

Supplementary Figure S15. Quantitative analysis of corrected total tissue fluorescence (CTTF). The cartoons schematically illustrate the way of randomly selecting five individual circular areas circumferentially along the wall of blood vessels.

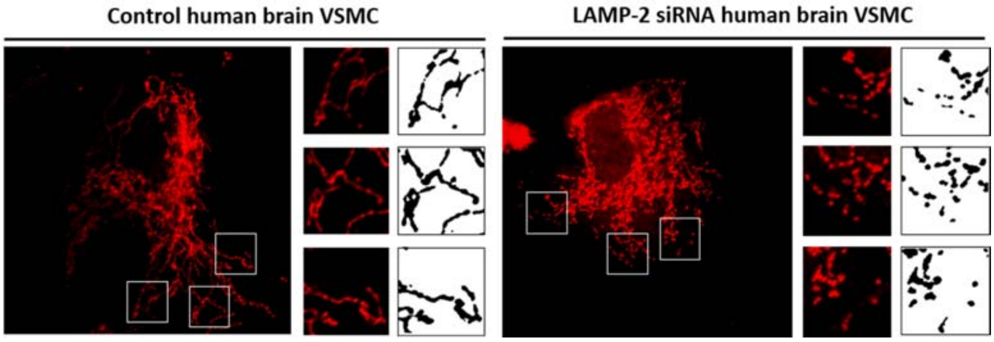

Supplementary Figure S16. Analysis of mitochondrial dynamics. Live-cultured human brain VSMC, under either scramble siRNA (control) or LAMP-2 siRNA-treated condition, were labelled with MitoTracker (*red*) for 20 min at 37°C. Cells were then fixed by 2% paraformaldehyde, and were observed under the confocal microscope. Control cells showed elongated mitochondrial network, whereas LAMP-2–deficient cells contained only a poorly organized network with short and fragmented pieces of mitochondria. By using the Image J software, the density and area of mitochondria were quantitated within at least three distinct areas randomly chosen.

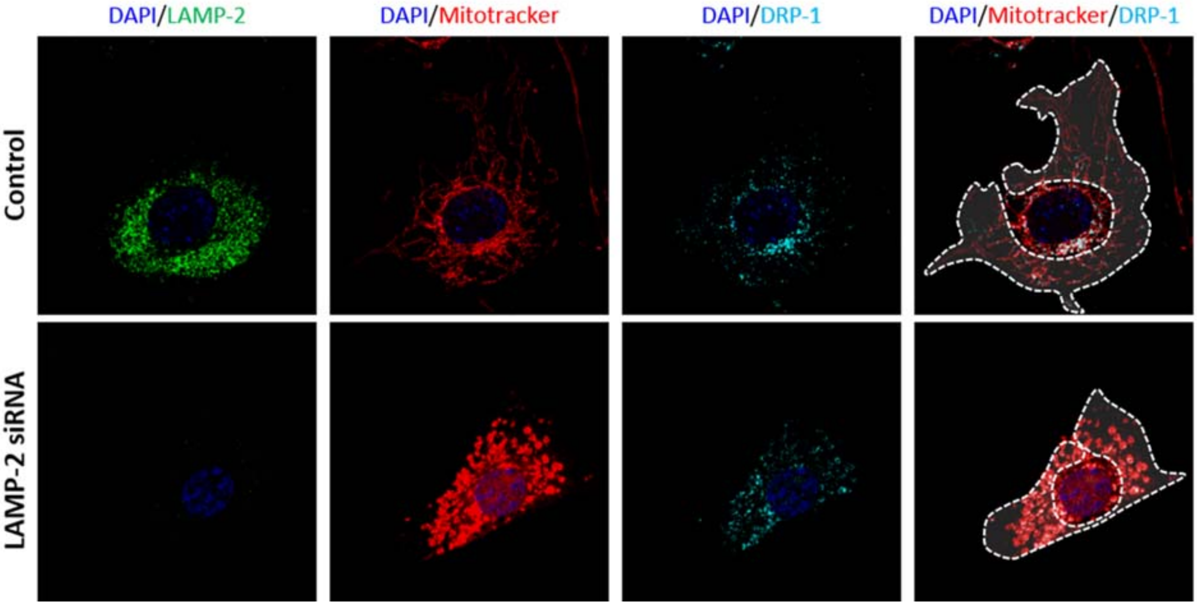

Supplementary Figure S17. Analysis of DRP-1 recruitment to mitochondria. After preloading with MitoTracker Red CMXRos *(red)*, cells were sequentially probed with rabbit monoclonal anti-DRP1 antibody *(turquoise)* and then mouse monoclonal anti-LAMP-2 antibody *(green)*. Colocalization between MitoTracker and DRP-1 was quantified using the software of ZEISS LSM 510 META for 20 cells from each group, control and LAMP-2 siRNA cells. The peripheral cytoplasmic region *(transparently white area)* was selected to quantitate the extent of colocalization.

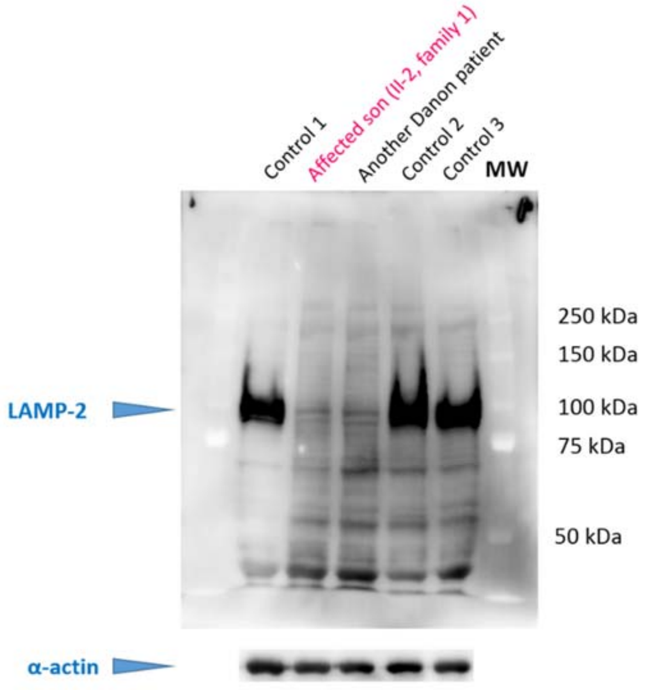

Supplementary Figure S18. Full unedited gel for Figure 1e. Western blot analysis of skeletal muscle in the affected son (II-2, family 1). α-actin was used as an internal control that ensures the equal loading. MW, molecular weight.

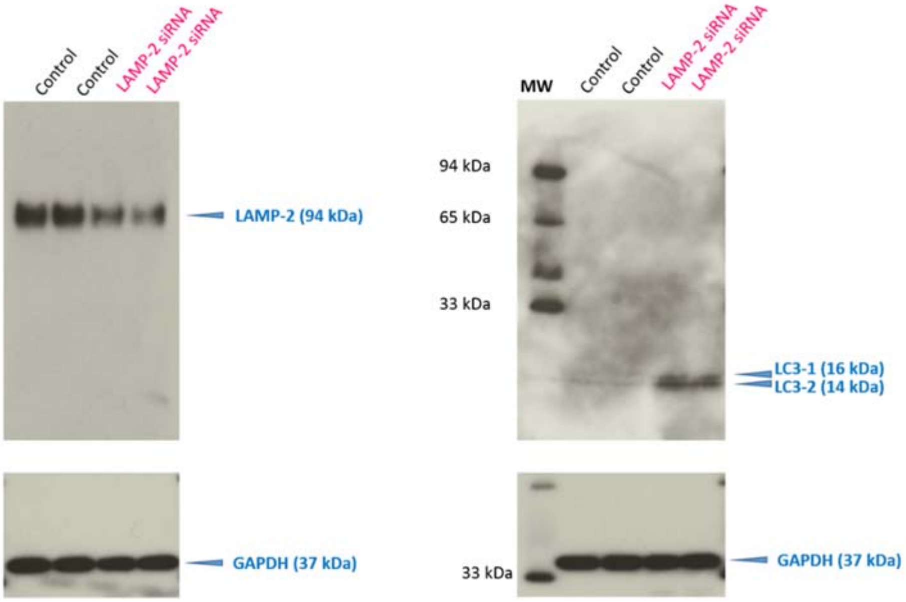

Supplementary Figure S19. Full unedited gel for Figure 4e. Western blot analysis for LAMP-2 protein *(left)* and LC3 *(right)* in human brain VSMC. Scramble siRNA (control) vs LAMP-2 siRNA-treated cells are compared. GAPDH was used as an internal control that ensures the equal loading. MW, molecular weight.

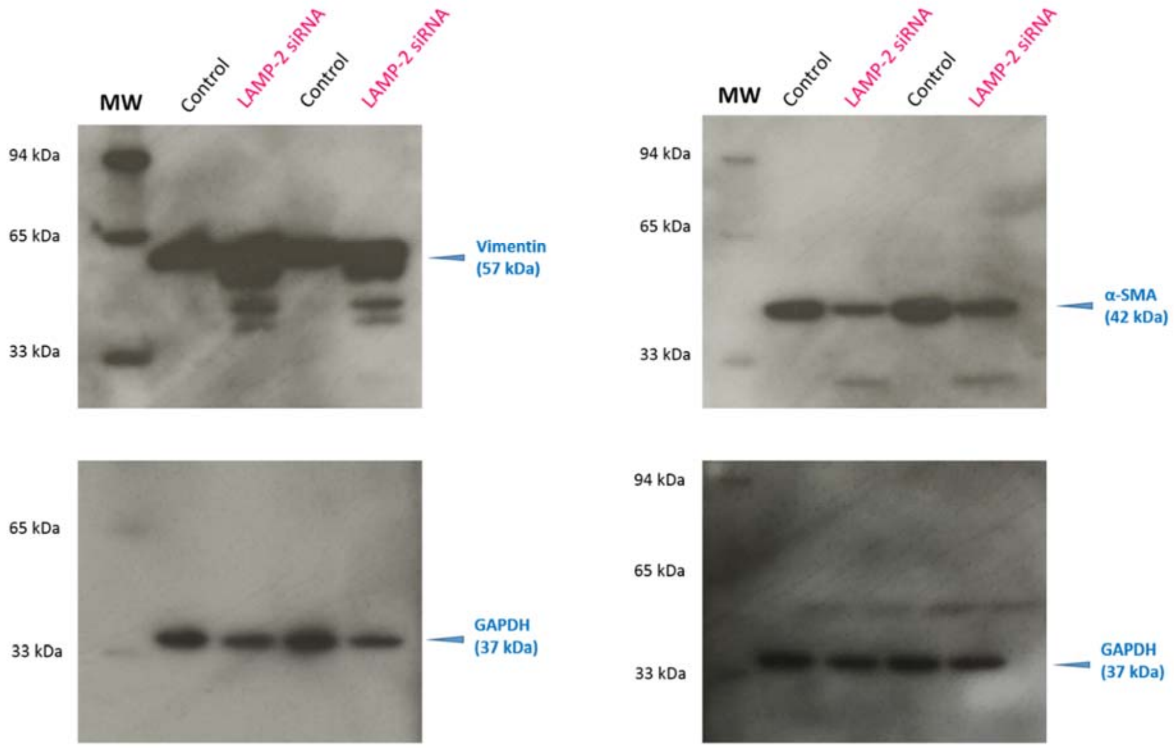

Supplementary Figure S20. Full unedited gel for Figure 5c. Western blot analysis for vimentin *(left)* and α-smooth muscle actin (α-SMA) *(right)* in human brain VSMC. Scramble siRNA (control) vs LAMP-2 siRNA-treated cells are compared. GAPDH was used as an internal control that ensures the equal loading. MW, molecular weight.

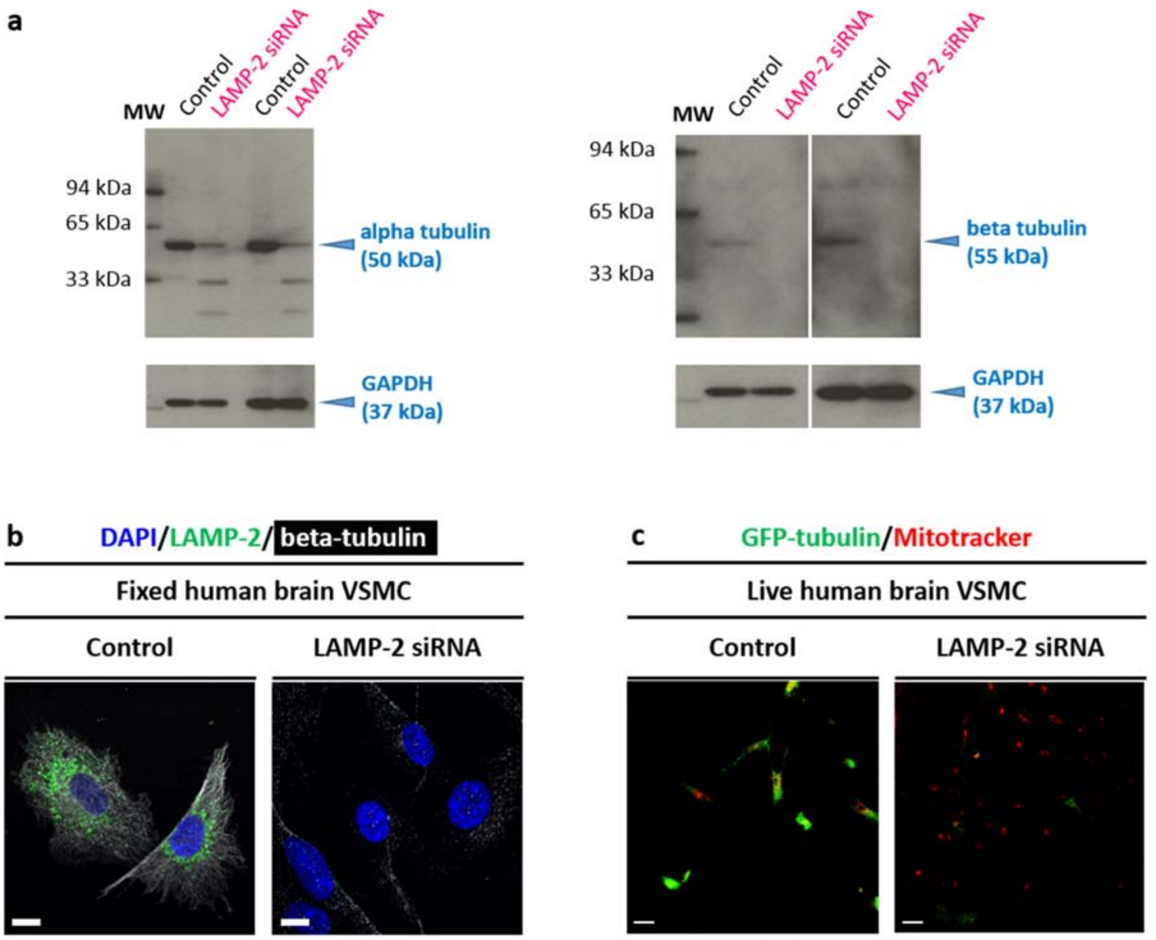

Supplementary Figure S21. Decreased expression of tubulin in LAMP-2–deficient human brain VSMC. (a) Western blot analysis for alpha-tubulin *(left)* and beta-tubulin *(right)* in human brain VSMC. Scramble siRNA (control) vs LAMP-2 siRNA-treated cells are compared. GAPDH was used as an internal control that ensures the equal loading. MW, molecular weight. **(b)** Immunostaining of beta-tubulin *(white)* in cultured human brain VSMC. Cells were fixed and probed with anti-LAMP-2 antibody *(green)* before observed under the confocal microscope. Nuclei were visualized by DAPI *(blue)*. Scale bars: 10 µm. **(c)** Live cell-imaging of cultured human brain VSMC. After 24 hours of transfection, cells were labelled with CellLight GFP-Tubulin *(green)*. On the day of the experiment, cells were loaded with MitoTracker Red CMXRos *(red)* for 20 min, and were recovered with growth media at 37°C before analysed under the confocal microscope. Scale bars: 50 µm.

Supplementary Tables

	Heterozygous mother (I-1)	Hemizygous son (II-2)
Current age	50 year-old	16 year-old
Cardinal manifestations	HCM, stroke (47 year-old)	HCM, myopathy, intellectual disability
**Cardiomyopathy**	(+) HCM	(+) HCM
Age at diagnosis for CVD, y	47	16
NYHA functional class	I	I
Cardiac magnetic resonance[†]		
Left ventricular mass, g (NR)	158 (70-142)	218.9 (107-187)
Ejection fraction, % (NR)	46.6 (58-76)	63.6 (57-75)
End diastolic volume, ml (NR)	118 (90-174)	99.7 (119-203)
End systolic volume, ml (NR)	63 (25-63)	36.6 (33-77)
BNP, pg/ml (NR: 0-18.4)	155	463
**Skeletal muscle weakness**	(–)	(+)
CK, U/L (NR: 43-165)	104	2632
**Central nervous system abnormalities**		
Intellectual disability	(–)	(+)
Brain MRA (age of years at examination)	Diffuse narrowing of cerebral arteries M2 (47)	Normal (16)
Brain MRI (age of years at examination)	Old infarction around the left putamen (50)	Normal cortical development in the absence of ischemic lesions (16)
**Liver dysfunction**	(–)	(+)
ALT, U/L (NR: 4-44)	25	441
AST, U/L (NR: 8-38)	48	403
**Lipid profile**		
Triglyceride, mg/dl (NR: 36-150)	82	122
HDL-c, mg/dl (NR: ≥ 40)	52	50
LDL-c, mg/dl (NR: 65-139)	85.6	115

Supplementary Table S1. Clinical features and laboratory findings in two patients of the first family with *LAMP2* mutation. The male proband had a hypertrophic cardiomyopathy, skeletal myopathy, intellectual disability, and liver functions, consistent with the diagnosis of a typical full-blown Danon disease. On the other hand, his mother developed young-onset stroke at age of 47 but recovered without leaving any motor and psychological deficits. The other overt clinical manifestation is cardiomyopathy only, of which the severity is similar to that of son. She had no abnormalities in skeletal muscle function, intellectual ability, and liver functions. ALT, alanine transaminase; AST, aspartate transaminase; BNP, B-type natriuretic peptide; CK, creatine kinase; CVD, cardiovascular diseases; HCM, hypertrophic cardiomyopathy; MRA, magnetic resonance angiography; MRI, magnetic resonance imaging; NR, normal range; NYHA, New York Heart Association[9]. †Normal ranges of left ventricular parameters are adapted from Kawel-Boehm *et al*[10].

Lamp2 genotypes	Age† (months)	Muscular arteries		
		Cerebral artery	Coronary artery	Femoral artery
		**Knockout**		
y/–	24	(–)	(–)	(+)
y/–	16	(+)	(–)	(+)
y/–	16	(–)	(–)	(–)
–/–	18	(–)	(–)	(–)
–/–	16	(–)	(–)	(+)
–/–	13	(–)	(–)	(+)
–/–	9	(–)	(–)	(+)
–/–	9	(–)	(–)	(+)
		Heterozygous		
+/–	24	(–)	(–)	(+)
+/–	20	(–)	(–)	(+)
+/–	18	(–)	(+)	(–)
+/–	9	(–)	(–)	(–)
		Wild-type		
+/+	24	(–)	(–)	(–)
+/+	24	(–)	(–)	(–)
+/+	18	(–)	(–)	(–)
+/+	18	(–)	(–)	(–)
+/+	18	(–)	(–)	(–)
y/+	19	(–)	(–)	(–)
y/+	19	(–)	(–)	(–)
y/+	19	(–)	(–)	(–)
y/+	16	(–)	(–)	(–)
y/+	16	(–)	(–)	(–)
y/+	16	(–)	(–)	(–)
y/+	9	(–)	(–)	(–)

Supplementary Table S2. Genotypes and frequency of vasculopathy in muscular arteries of LAMP-2–deficient mice. †We chose the mice with the age ranging from 9 to 24 months, which are equivalent to those at 28 to 75 years of human[11], assuming that the aging may contribute to the pathogenesis of the vascular lesions. (–), the absence of thickened media of arteries, (+), the presence of thickened media of arteries. Genotypes are indicated by the symbols: +, *Lamp2* wild-type allele; –, *Lamp2* null allele; and y, Y chromosome.

Parameter	Wild-type *(n = 12)*	LAMP-2–deficient mice *(n = 12)*	
	Female *Lamp2*$^{+/+}$ *(n = 5)*<br>Male *Lamp2*$^{y/+}$ *(n = 7)*	Female heterozygous<br>*Lamp2*$^{+/–}$ *(n = 4)*	Female knockout *Lamp2*$^{–/–}$ *(n = 5)*<br>Male knockout *Lamp2*$^{y/–}$ *(n = 3)*
**Physical features**			
Age of month	18.0 ± 3.9	17.8 ± 6.3	15.1 ± 4.9
Body weight, g	35.1 ± 5.2	32.0 ± 4.3	26.5 ± 3.3**
Heart weight†, g	0.23 ± 0.04	0.22 ± 0.04	0.24 ± 0.03
**Morphometric parameters of femoral arteries**			
Media thickness, µm	15.0 ± 2.1	30.6 ± 6.9**	38.1 ± 11.6***
Medial CSA, 10^3 x μm^2	8.9 ± 3.1	13.7 ± 3.4*	18.1 ± 2.8***
Lumen diameter, µm	123.9 ± 7.7	106.7 ± 18.8	102.8 ± 16.2**
Luminal area, 10^3 x μm^2	12.1 ± 1.5	8.8 ± 2.9*	8.4 ± 2.4**
M/L, %	12.1 ± 1.8	30.1 ± 11.2**	38.6 ± 14.1***

Supplementary Table S3. General physical features and morphometric analysis of femoral arteries of LAMP-2–deficient mice. Data are indicated as mean ± SD. Data were analyzed by two-sided one-way ANOVA followed by the post hoc Tukey test. * $P < 0.05$ vs. wild-type; ** $P < 0.01$ vs. wild-type; ***$P < 0.001$ vs. wild-type. †Heart weights did not significantly differ among the three subgroups. It is therefore reasonable to assume that the morphometric changes of femoral arteries in LAMP-2–deficient mice primarily reflect the biological dysfunction intrinsic to VSMC, but not to the secondary responses to altered hemodynamics or circulation problems. CSA, cross-sectional area; M/L, media thickness to lumen diameter ratio.

**Parameter**	**Female wild-type *Lamp2*$^{+/+}$ (*n = 5*)**	**Female heterozygous *Lamp2*$^{+/-}$ (*n = 4*)**
**Physical features**		
Age of month	20.4 ± 3.3	17.8 ± 6.3
Body weight, g	31.7 ± 6.0	32.0 ± 4.3
Heart weight†, g	0.22 ± 0.04	0.22 ± 0.04
**Morphometric parameters of femoral arteries**		
Media thickness, µm	14.76 ± 1.8	30.6 ± 6.9**
Medial CSA, 10^3 x µm^2	7.6 ± 3.0	13.7 ± 3.4*
Lumen diameter, µm	122.7 ± 8.7	106.7 ± 18.8
Luminal area, 10^3 x µm^2	11.86 ± 1.7	8.8 ± 2.9
M/L, %	12.14 ± 2.1	30.1 ± 11.2*

Supplementary Table S4. Comparison between female wild-type *Lamp2*$^{+/+}$ and female heterozygous *Lamp2*$^{+/-}$ mice. Data are indicated as mean ± SD. Data were analyzed by two-tailed *Student's* t-tests. * $P < 0.05$ vs. wild-type; ** $P < 0.01$ vs. wild-type. The morphometric analysis with the femoral arteries revealed the tendency that the vascular wall is thicker, while the lumen is narrower, in female heterozygous *Lamp2*$^{+/-}$ mice than in female wild-type mice. CSA, cross-sectional area; M/L, media thickness to lumen diameter ratio.

1st Antibodies			2nd Antibodies		
**Name**	**Source (cat. number)**	**Working dilution**	**Name**	**Source (cat. number)**	**Working dilution**
**Immunofluorescence for frozen tissues**					
Rabbit monoclonal anti-LC3A/B (D3U4C) XP	Cell Signaling (#12741)	1:500	Goat anti-rabbit (Alexa Fluor 594)	Abcam (ab150088)	1:500
Rabbit monoclonal anti-α smooth muscle actin	Abcam (ab32575)	1:500	Goat anti-rabbit (Alexa Fluor 488)	Abcam (ab150085)	1:500
Rabbit monoclonal anti-vimentin (D21H3) XP	Cell Signaling (#5741)	1:200	Goat anti-rabbit (Alexa Fluor 488)	Abcam (ab150085)	1:500
Rat monoclonal anti-CD31	GeneTex (GTX54379)	1:500	Donkey anti-rat (Alexa Fluor 594)	Invitrogen (A-21209)	1:500
Goat polyclonal anti-α smooth muscle actin	Abcam (ab21027)	1:500	Chicken anti-goat IgG (Alexa Fluor 647)	Invitrogen (A-21469)	1:500
**Immunocytochemistry**					
Mouse monoclonal anti-LAMP-2/CD107b	NovusBio (NBP2-22217)	1:200	Goat anti-mouse (Alexa Fluor 488)	Abcam (ab150117)	1:500
			Rabbit anti-mouse (Alexa Fluor 647)	Abcam (ab150127)	1:500
Rabbit monoclonal anti-LC3A/B (D3U4C) XP	Cell Signaling (12741S)	1:500	Goat anti-rabbit (Alexa Fluor 594)	Abcam (ab150088)	1:500
Rabbit monoclonal anti-α smooth muscle actin	Abcam (ab32575)	1:200	Goat anti-rabbit (Alexa Fluor 594)	Abcam (ab150088)	1:500
Rabbit monoclonal anti-vimentin (D21H3) XP	Cell Signaling (#5741)	1:200	Goat anti-rabbit (Alexa Fluor 594)	Abcam (ab150088)	1:500
Rabbit monoclonal anti-DRP-1	Abcam (ab184247)	1:250	Sheep anti-rabbit (Alexa Fluor 650)	Abcam (ab96926)	1:500
Rabbit polyclonal anti-β tubulin (H-235)	Santa Cruz (sc-9104)	1:200)	Goat anti-rabbit (Alexa Fluor 594)	Abcam (ab150088)	1:500
**Western blot**					
Mouse monoclonal anti-LAMP-2/CD107b	NovusBio (NBP2-22217)	1:1000	ECL peroxidase labelled anti-mouse	GE Healthcare (NA931VS)	1:10,000
Rabbit monoclonal anti-LC3A/B (D3U4C) XP	Cell Signaling (12741S)	1:1000	ECL peroxidase labelled anti-rabbit	GE Healthcare (NA934VS)	1:10,000
Rabbit monoclonal anti-α smooth muscle actin	Abcam (ab32575)	1:1000	ECL peroxidase labelled anti-rabbit	GE Healthcare (NA934VS)	1:10,000
Rabbit monoclonal anti-vimentin (D21H3) XP	Cell Signaling (#5741)	1:1000	ECL peroxidase labelled anti-rabbit	GE Healthcare (NA934VS)	1:10,000
Mouse monoclonal anti-GAPDH	Wako (014-25524)	1:1000	ECL peroxidase labelled anti-mouse	GE Healthcare (NA931VS)	1:10,000
Rabbit polyclonal anti-α actin	Kantokagaku (01867-96)	1:1000	ECL peroxidase labelled anti-rabbit	GE Healthcare (NA934VS)	1:10,000
Rabbit polyclonal anti-β tubulin (H-235)	Santa Cruz (sc-9104)	1:1000	ECL peroxidase labelled anti-rabbit	GE Healthcare (NA934VS)	1:10,000
Mouse monoclonal anti-α tubulin (DM1A)	Abcam (ab7291)	1:5000	ECL peroxidase labelled anti-mouse	GE Healthcare (NA931VS)	1:10,000

Supplementary Table S5. List of first antibodies and corresponding second antibodies.

Supplementary References

1 Sugie, K. *et al.* Autophagic vacuoles with sarcolemmal features delineate Danon disease and related myopathies. *Journal of neuropathology and experimental neurology* **64**, 513-522 (2005).

2 Endo, Y., Furuta, A. & Nishino, I. Danon disease: a phenotypic expression of LAMP-2 deficiency. *Acta neuropathologica* **129**, 391-398, doi:10.1007/s00401-015-1385-4 (2015).

3 Yang, Z. & Klionsky, D. J. Eaten alive: a history of macroautophagy. *Nature cell biology* **12**, 814-822, doi:10.1038/ncb0910-814 (2010).

4 Maron, B. J. *et al.* Clinical outcome and phenotypic expression in LAMP2 cardiomyopathy. *Jama* **301**, 1253-1259, doi:10.1001/jama.2009.371 (2009).

5 Eskelinen, E. L. Roles of LAMP-1 and LAMP-2 in lysosome biogenesis and autophagy. *Molecular aspects of medicine* **27**, 495-502, doi:10.1016/j.mam.2006.08.005 (2006).

6 Carrel, L. & Willard, H. F. X-inactivation profile reveals extensive variability in X-linked gene expression in females. *Nature* **434**, 400-404, doi:10.1038/nature03479 (2005).

7 Vilchez, D., Saez, I. & Dillin, A. The role of protein clearance mechanisms in organismal ageing and age-related diseases. *Nature communications* **5**, 5659, doi:10.1038/ncomms6659 (2014).

8 Rzucidlo, E. M., Martin, K. A. & Powell, R. J. Regulation of vascular smooth muscle cell differentiation. *Journal of vascular surgery* **45 Suppl A**, A25-32, doi:10.1016/j.jvs.2007.03.001 (2007).

9 Yancy, C. W. *et al.* 2013 ACCF/AHA guideline for the management of heart failure: a report of the American College of Cardiology Foundation/American Heart Association Task Force on practice guidelines. *Circulation* **128**, e240-327, doi:10.1161/CIR.0b013e31829e8776 (2013).

10 Kawel-Boehm, N. *et al.* Normal values for cardiovascular magnetic resonance in adults and children. *Journal of cardiovascular magnetic resonance : official journal of the Society for Cardiovascular Magnetic Resonance* **17**, 29, doi:10.1186/s12968-015-0111-7 (2015).

11 Dutta, S. & Sengupta, P. Men and mice: Relating their ages. *Life sciences* **152**, 244-248, doi:10.1016/j.lfs.2015.10.025 (2016).
